# Evaluation of inhibitive corrosion potential of symmetrical hydrazine derivatives containing nitrophenyl moiety in 1M HCl for C38 steel: experimental and theoretical studies

**DOI:** 10.1016/j.heliyon.2022.e09087

**Published:** 2022-03-15

**Authors:** Zouhair Lakbaibi, Mohamed Damej, Abdu Molhi, Mohammed Benmessaoud, Said Tighadouini, Adil Jaafar, Tariq Benabbouha, Abdeselam Ansari, Anas Driouich, Mohamed Tabyaoui

**Affiliations:** a Laboratory of Molecular Chemistry, Materials and Environment, Department of Chemistry, Multidisciplinary Faculty, Mohamed first University, BP 300 Selouane 62700, Nador, Morocco; b Environment, Materials and Sustainable Development Team-CERNE2D, High School of Technology, Mohammed V University, Rabat, Morocco; c Laboratory of Spectroscopy, Molecular Modelling Materials, Nanomaterials Water and Environment, Faculty of Sciences, Mohammed V University, Rabat, Morocco; d Laboratory of Organic Synthesis, Extraction and Valorization, Faculty of Sciences Ain Chock, Hassan II University, EL Jadida Road, Km 2, BP: 5366, Casablanca, Morocco; e Higher School of Education and Training of Berrechid, Hassan 1^st^ University Settat, Morocco; f Thermodynamic, Catalysis and Surface Team, Faculty of Sciences, Chouaib Doukkali University, BP:24 000, El Jadida, Morocco; g Regional Center for Education and Training Professions, Al Mouzdalifa Street, Marrakech 40000, Morocco; h Laboratory of chemical engineering and environment, Faculty of Sciences and Techniques, Hassan II University, Mohammedia, Morocco; i Laboratory of Materials, Nanoparticles and Environment, Mohammed V University, Faculty of Sciences, BP 1014, Ibn Batouta Avenue, Rabat, Morocco

**Keywords:** Hydrazine derivatives, Nitrophenyl, Green, C38 steel corrosion, DFT, QTAIM, Molecular dynamics simulation

## Abstract

The exploitation of cost-effective, sustainable, green and efficient compounds is a renewed science and a demanding mission for today's chemists and technologists. In this view, the inhibitive corrosion properties of some hydrazine derivatives named (1*E*,2*E*)-bis(1-(2-nitrophenyl)ethylidene)hydrazine (**SSBO**), (1*E*,2*E*)-bis(1-(3-nitrophenyl)ethylidene)hydrazine (**SSBM**) and (1*E*,2*E*)-bis(1-(4-nitrophenyl)ethylidene)hydrazine (**SSBP**) on the C38 steel corrosion in 1M HCl media has been evaluated by different techniques like electrochemical impedance spectroscopy (EIS), potentiodynamic polarization (PDP), scanning electron microscopy (SEM) and energy-dispersive X-ray spectroscopy. The EIS results showed that **SSBM** is the greatest inhibitor (η>93%) among the three tested compounds. The **SSBM** gives considerable inhibition efficiency against corrosion of steel compared to the previous studies. The PDP curves indicated that the studied inhibitors were a mixed-type inhibitor with a predominantly cathodic control. Quantum calculations of some descriptors derived from the density functional theory (DFT), the transition state theory (TST), the quantum theory of atoms in molecules (QTAIM) and molecular dynamics simulation have delivered helpful information regarding electron transfer and mechanism during adsorption of inhibitors on C38 steel surface.

## 1 Introduction

C38 steel is a material extensively used in many engineering domains, which due to its low cost and high availability for the building of several industrial materials [1]. The foremost problem of applying C38 steel in the industry is its destruction in different aggressive environments, particularly in acidic solutions [2]. Unfortunately, acid solutions are frequently used in industry for cleaning, descaling, oil-well acidification, additives, and petroleum processes [3]. Generally, the corrosion phenomenon in C38 steel metal is an electrochemical reaction that starts with transfer of electron zero-valent Fe atom of C38 steel to an external electron acceptor, conducting to the release of the metal ions and degradation of metal [4]. Inhibited acid solutions are mostly applied to protect metals from corrosion in acidic media [5]. These inhibitors reduce the corrosion rate of the metal by retarding anodic and/or cathodic reactions, decreasing the movement and/or diffusion of ions and growing electrical resistance of the metal [6], [7]. Additionally, most organic inhibitors that were used against corrosion can be adsorbed on the metal surface via e-poor and/or e-rich sites such as heteroatoms, unsaturated bonds or aromatic rings, lone-pair and/or *π* electrons, orbitals π*, reactive functional groups, and inversely charged sites relative to the charged metal surface [8], [9]. Nowadays, innovative studies are going on for the sustainable ecosystem development of so-called “eco-friendly” corrosion inhibitors by the use of efficient and inexpensive compounds with a weak or nothing negative environmental impact [7].

**Figure 1:**
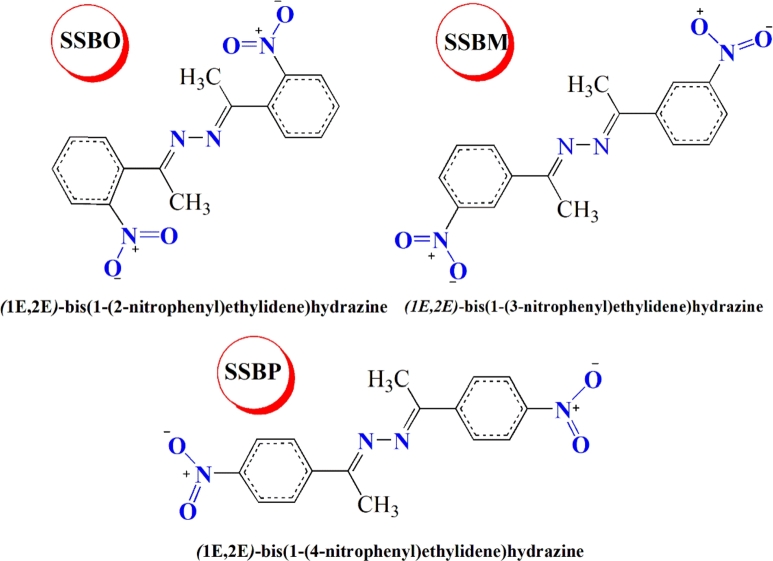
Molecular structures of **SSBs.**

In view of these recommendations we have exploited some symmetrical hydrazine derivatives containing nitrophenyl moiety, namely (1*E*,2*E*)-bis(1-(2-nitrophenyl)ethylidene)hydrazine (**SSBO**), (1*E*,2*E*)-bis(1-(3-nitrophenyl)ethylidene)hydrazine (**SSBM**) and (1*E*,2*E*)-bis(1-(4-nitrophenyl)ethylidene)hydrazine (**SSBP**) to explore their inhibitive corrosion properties for C38 in molar hydrochloric solution using electrochemical impedance spectroscopy (EIS), potentiodynamic polarization (PDP), and surface analysis using scanning electron microscopy with energy-dispersive X-ray spectroscopy (SEM-EDS) and element mapping via electronic-microscopy horizontal scanning test (EM). Then, quantum calculations based on the density functional theory (DFT), quantum theory of atoms in molecules (QTAIM), molecular electrostatic potential surface (MEP) analysis, transition state theory (TST) and molecular dynamics (MD) simulation were achieved. These three theoretical approaches have been proven to be very effective in finding new physic-chemical information on the adsorption mechanism. These theoretical studies allow investigating and establishing a relationship between the efficiency of corrosion inhibition of the **SSBs** compounds (i.e., **SSBO**, **SSBM** and **SSBP**) and the electron density distributed within each one of them. Also, these techniques will give more information regarding the effect of nitro group on molecular structure behavior of three compounds (**SSBs**) presented in Fig. 1. Previously studies were carried about inhibitive corrosion of other hydrazine derivatives which showed inhibition efficiency ranged from 62.40 to 86.70% at optimum concentration of 1mM in molar hydrochloric acid [10], [11], [12]. One compound of our tested hydrazine molecules gives best inhibition efficiency of 93.04% at the same optimum concentration.

The choice of these hydrazine derivatives (**SSBs**) to test their anti-corrosive properties is based on the following cautions (a) their synthesis process easy and not expensive, (b) they are formed with high yield, (c) have many biological activities and excellent fluorescent chemosensors for metal ions [13], (d) they are no poisonous, (e) contain heteroatoms (N and O), (f) contain ortho-, meta- or para-nitro group (with electron-withdrawing) leading to the partial charges on carbon chain of aromatic ring, (g) they are *π*-conjugated systems with 2,3-diaza group (R-C=N-N=C-R), (h) possess methyl group (with electron-donor) which ensure availability of electron density around nitrogen atoms of 2,3-diaza group, (i) they are symmetrical systems [13], [14]. The considerations (d), (e), (f), (g), (h) and (i) offer the studied compounds a high ability to form covalent or/and non-covalent bonds with C38 steel and thus forming a protective layer against corrosion. According to an extensive bibliographic study, until this date, no work has been done on these compounds as corrosion inhibitors.

## 2 Experimental details

### 2.1 C38 steel samples and solutions

The metal used is C38 steel (CS), its composition with masse percentage is as follows: C 0.370%, Si 0.230%, Mn 0.680%, S 0.016%, Cr 0.077%, Ti 0.011%, Ni 0.059%, Co 0.009%, Cu 0.160% and Fe 98.307% [1]. The CS area of 1 cm^2^ was prepared and insulated with a resin. Corrosion media of 1M HCl solution prepared from analytical grade 37% HCl by dilution with distilled water.

### 2.2 Inhibitors

The tested hydrazine derivatives (**SSBs**) were resynthesized with high yields according to the protocol defined in the following reference [14]. All details about the characterization of **SSBs** products are defined in the same reference. The molecular structure, acronyms and IUPAC names of concerned compounds are given in Fig. 1.

### 2.3 Electrochemical experiments

Electrochemical tests were performed using a potentiostat PGZ101 and a three-electrode cell, controlled by a computer machine equipped with Voltamaster4. The CS, the platinum and the electrode with saturated calomel Ag/AgCl/KCl (SCE) are used respectively as working electrode, counter electrode and reference electrode. The electrochemical tests were carried out in 1M HCl solution without and with different concentrations of inhibitor (0.05 mM, 0.1 mM, 0.5 mM, and 1 mM). Nyquist and Bode curves were plotted after the 1 hour immersion in the corrosive solution in the absence and in the presence of different concentrations of the inhibitor using an Ac signal with amplitude of 10 mV peak to peak and a frequency range of 100 kHz to 10 mHz [8]. Equivalent circuit was obtained by the fitting of the experimental curves using Ec-Lab software. The PDP curves were logged from −800 to −100 mV at a scan rate of 0.5 mV/s [8]. To guarantee the reproducibility of electrochemical results, all EIS and PDP measurements were repeated three times. The Ec-Lab10.36 and OriginPro8 software were used to analyze results from EIS and PDP. The fitting errors did not surpass 10% and the $\chi^{2}$ was below 10^−3^.

### 2.4 Surface characterization

The pre-treated CS surface was analyzed before and after its emersion in acidic media in the presence and absence of 1mM of **SSBs** for 24 hours at 308 K with digital microscope imaging *TESCAN* model equipped with a chemical analysis system with an accelerating voltage of 15 kV allowing penetration of the electron beam into the material and leading to the realization of extreme surface chemical analysis.

### 2.5 Quantum calculation details

Full geometry optimizations of all structures were carried out using the DFT theory and the B3LYP functional coupled together to the 6-311G++(2d,2p) basis set for the following atoms H (hydrogen), C (carbon), N (nitrogen) and O (oxygen). However, for the iron atoms (Fe), the basis set 6-311G++(2d,2p) was replaced by another one that is more appropriate its name is abbreviated as LanL2DZ. All DFT calculations were achieved under an implicit solvation model (SMD) using Gaussian 09 Revision D-01 [15], [16]. The DFT, QTAIM, MESP and TST theories were used to give more supplementary information regarding the molecular structure of inhibitor and confirm the results obtained experimentally. Several quantum chemical descriptors that indicate structural characteristics of organic inhibitors (**SSBs** and **SSBH+**), such as E_HO(inh)_ (energy of the highest occupied molecular orbital), E_BV(inh)_, (energy of the lowest unoccupied molecular orbital), Fermi level energy (FEL) of Fe(110) and so on, were obtained [17]. DFT also provides a convenient theoretical framework for calculating global and local indices that describe chemical reactivity of chemical species quantitatively. The energy gap (ΔE_(inh)_), energy gap (ΔE_(inh-Fe)_), chemical potential *μ*, electronegativity (*χ*), chemical hardness (*η*) and chemical softness (*σ*), which make it possible to evaluate the tendency of an atomic site to acquire electrons, these descriptors are calculated using the following equations (1)–(5), respectively [18]. Therefore, the electrophilic index (*ω*) is a popular quantum descriptor that defines the capacity of a compound to receive electrons from an acceptor chemical species. The descriptor *ω* can be calculated by the following equation (6) [18]. Besides, the flow of electrons (ΔN_(110)_) moved from (to) the inhibitor to (from) the metal surface was calculated according to the following equation (7).

(1) $$\Delta E_{\left(\mathrm{inh}\right)}=E_{\mathrm{BV}\left(\mathrm{inh}\right)}-E_{\mathrm{HO}\left(\mathrm{inh}\right)}$$

(2) $$\Delta E_{\left(\mathrm{inh}-\mathrm{Fe}\right)}=E_{\mathrm{HO}\left(\mathrm{inh}\right)}-\mathrm{FEL}$$

where FEL = $\frac{E_{\mathrm{HO}(\mathrm{Fe}(110)}+E_{\mathrm{BV}(\mathrm{Fe}(110)}}{2}$)

(3) $$\mu =-\chi =\frac{E_{\mathrm{HO}\left(\mathrm{inh}\right)}+E_{\mathrm{BV}(\mathrm{inh})}}{2}$$

(4) $$\eta =\frac{E_{\mathrm{BV}\left(\mathrm{inh}\right)}-E_{\mathrm{HO}(\mathrm{inh})}}{2}$$

(5) $$\sigma =\frac{1}{\eta }=\frac{2}{E_{\mathrm{BV}\left(\mathrm{inh}\right)}-E_{\mathrm{HO}(\mathrm{inh})}}$$

(6) $$\omega =\frac{\mu^{2}}{2\eta }$$

(7) $$\Delta N_{\left(110\right)}=\frac{\left(\chi_{\mathrm{Fe}(110)}-\chi_{\mathrm{inh}}\right)}{2(\eta_{\mathrm{Fe}(110)}+\eta_{\mathrm{inh}})}$$

The active sites existing in an organic molecule of inhibitor are determined by analyzing the local reactivity indices such as Parr functions *P*− (electrophilic attack) and *P*+ (nucleophilic attack) based on the Mulliken atomic spin density (MASD) calculations [19] and using Fukui functions *f*− and *f*+ calculated in terms of natural population analysis (NPA). The high value of *P*+ or *f*+ measures the change in electron density at an atomic site when the molecule belongs to receive an extra electron and *P*− or *f*− measures the change in electron density at an atomic site when the molecule belongs to has lost an extra electron. The local reactivity was also evaluated basing on the HOMO and LUMO shapes, molecular electrostatic potential surface (MEP) and non-shared electron density (NSED), which calculated according to the quantum theory of atom in molecules (QTAIM) [20], [21], [22]. Moreover, in order to contribute more understanding of the local reactivity associated with the studied molecules, dual local functions that are defined for an atomic site k as dual local Fukui $\Delta f_{k}$ (the local nucleophilic Fukui index *f*− minus electrophilic Fukui index *f*+), dual local softness $\Delta \sigma_{k}$ (the nucleophilic local softness *σ*− minus the local softness *σ*+), and the philicity $\Delta \omega_{k}$ (the local nucleophilic index *ω*− minus the local electrophilic index *ω*+) are calculated and discussed [18].

To investigate non-covalent interactions (NCI) which can characterize inhibitor structure, an empirical dispersion GD3 scheme by Grimme was added to the B3LYP functional at DFT theory [23], [24]. Unlike B3LYP, the corrected functional B3LYP-GD3 appears to be reliable for investigating non-covalent interactions [23], [24]. The NCI calculations were performed using Multiwfn and VMD software [25]. In quantum chemistry, the NCI theory aimed to visualize qualitatively non-covalent interactions in 3D dimensions of the molecular space. Therefore, the visual NCI depiction arises from the isosurfaces of the reduced density gradient *S* which is colored by a scale of strength defined as follows: strong attractive interactions (blue), weak interactions (green) and strong repulsive interactions (red). The gradient *S* is expressed as a function of the electron density *ρ*(r) according to the following equation (8) [25]:

(8) $$S=\frac{1}{2{\left(3\pi^{2}\right)}^{1/3}}\frac{\left|\nabla \rho \right|}{\rho^{4/3}}$$

The frequency calculations of the stationary points were displayed to verify the number of imaginary frequencies *fi* (zero for global minima and one for TSs) [26]. The TSs of the corresponding complexes “inhibitor⋯Fe” were localized using transition state theory on the basis of second-order Gonzalez-Schlegel integration quadratic synchronous transit-guided quasi-Newton (QST2) approach using both B3LYP and B3LYP-GD3 functional at DFT method [27], [28]. The localization of TSs structures is subsequently followed by analysis IRC (intrinsic reaction coordinates (IRC) in order to check the energy profiles connecting each TS to both associated minima (reagents and products) [19], [29]. The global electronic density transfer (GEDT) at the TSs was calculated by the sum of the natural atomic charges (Q) of the atoms belonging to both donor (Q > 0) and acceptor (Q < 0) fragments (F) characterized the TSs structures. The direction of the electron density flux takes place from the donor fragment to the acceptor fragment one [30], [31].

The strength of coordination between active sites of inhibitor and iron atoms was evaluated by analyzing calculating the electronic configurations and calculating the second-order stabilization energy E^(2)^ calculated using NBO analysis. For each donor side NBO(i) and acceptor NBO (j), the E^(2)^ energy, which is associated with the electron density delocalization between donor and acceptor (i→j), is estimated by the following equation (9) [32]:

(9) $$E^{\left(2\right)}=\Delta E_{ij}=q_{i}\frac{{\left(F_{ij}\right)}^{2}}{\left(\varepsilon_{i}-\varepsilon_{j}\right)}$$

where q_i_ is the orbital occupancy, *ε*_i_, and *ε*_j_ are NBO diagonal elements (orbital energies), and F_ij_ is the off-diagonal NBO Fock matrix element.

### 2.6 MD simulation methodology

Pre-optimization of Fe (110) surface was carried using generalized gradient approximation (GGA) in the Dmol̂3 module with the polarization function DNP+ implanted in Material Studio 2016 (MSv.8.0) software package [33]. The simulation of the chemical species such as **SSBs**, 20 Chloride ions (20Cl-), 5 oxonium ions (5H3O+) and 500 water molecules (500 H2O) was carried out on a supercell containing 6 layers of iron atoms using forcite module integrated into MSv.8.0 software package. The calculation parameters for the MD simulations are gathered in Table 1 [33].

**Table 1:** Details on molecular dynamics calculations [33].

Simulation cell parameters	Convergence criteria	Dynamics parameters	Calculation code
Lattice: 3D triclinic	Algorithm Smart	Ensemble NVT	Force field
Cell size: a = 32.27 Å b = 32.27 Å c = 30.13 Å	Quality convergence: tolerance ultra-fine	Time step: 1 fs Simulation time: 400 ps Steps number: 5000	Compass
Iron atoms number: 1176	Energy convergence: 5 × 10^−5^ kcal/mol	Cutoff distance: 15.5 Å Thermostat Indersen Spline width: 1 Å Buffer width 0.5 Å	Pre-optimization of iron surface by GGA in the Dmol̂3 module with DNP+

## 3 Results and discussion

### 3.1 EIS measurements

**Figure 2:**
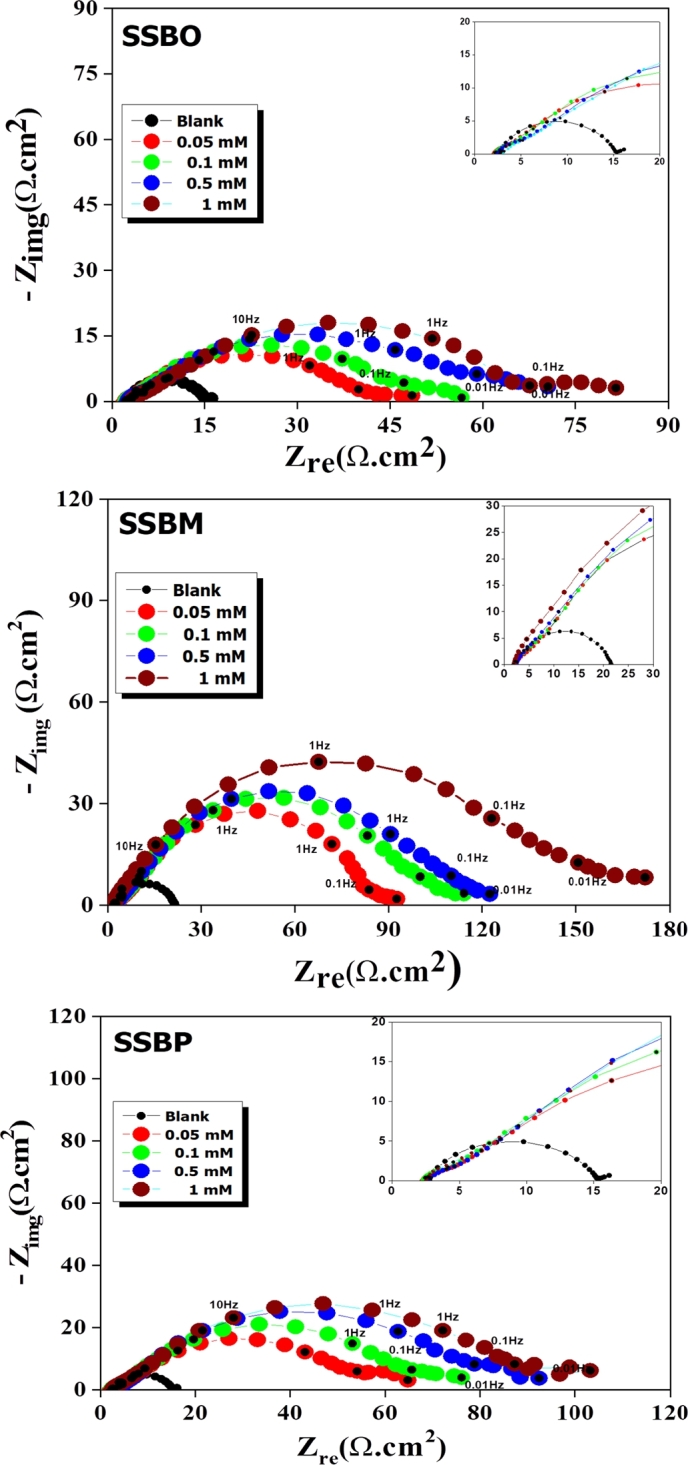
Nyquist plots for CS in 1M HCl solution without and with different concentrations of **SSBs** at 308 K.

**Figure 3:**
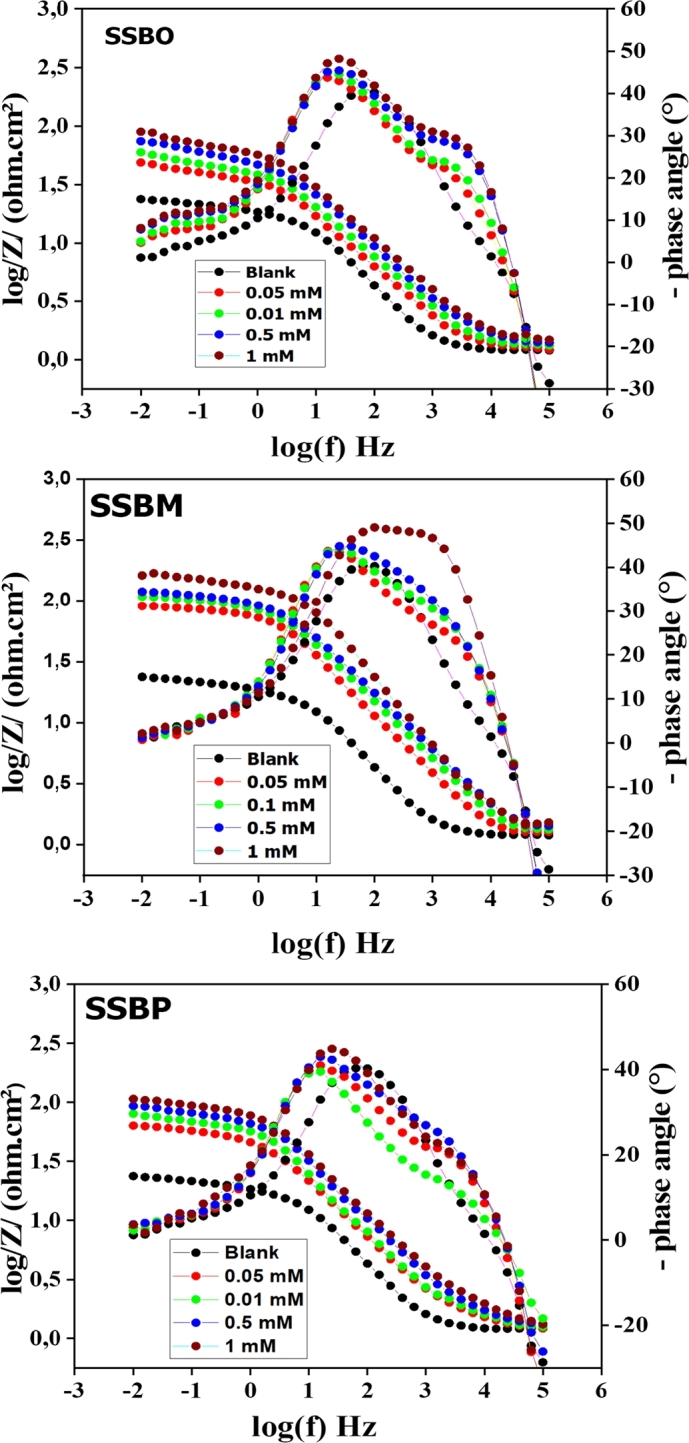
Bode and phase angle plots for CS in 1M HCl solution without and with different concentrations of **SSBs** at 308 K.

**Figure 4:**
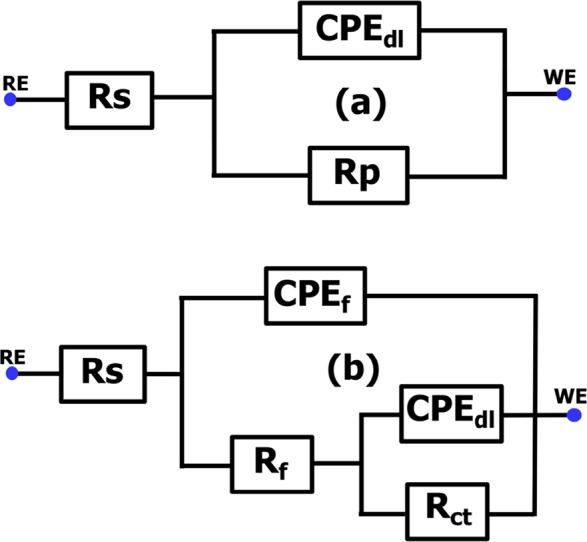
Proposed equivalent circuit model for the studied system: (a) without inhibitor; (b) with inhibitor.

Nyquist and Bod representation of EIS plots for CS in 1M HCl without and with inhibitor at various concentrations are shown in Fig. 2 and 3, respectively. As shown in Fig. 2, the loops are not perfect semicircles which indicate that the three compounds develop a non-perfect layers; this behavior can be attributed to the frequency dispersion, which is due to the roughness and inhomogeneity of the CS surface [8]. Furthermore, in the uninhibited solution, we note the appearance of a single capacitive loop, whereas in the presence of inhibitor, the shape and size of the Nyquist traces are different. Indeed, they present two superimposed capacitive loops. At high frequency a first capacitive loop was attributed to the formation of a film barrier by the inhibitor on the CS surface, while the second at low frequency was related to the charge transfer capacity [13], [34]. This result was confirmed through the analysis of Bode plots (presence of two-time constants) Fig. 3. Note that all three **SSBs** compounds exhibited the same electrochemical behavior exhibited the same electrochemical behavior. The equivalent circuit shown in Fig. 4 is used for modeling of impedance spectra of **SSBs** on C38 steel surface [35], [36]. The circuit is composed of solution resistance (*Rs*), inhibitor film resistance ($R_{f}$), charge transfer resistance ($R_{ct}$), and two constant phase elements such as the constant phase element of the double layer capacity (${\mathrm{CPE}}_{dl}$) and inhibitor film constant phase element (${\mathrm{CPE}}_{f}$,*n*) [8]. The constant phase element (*CPE*) is usually used to take into account the inhomogeneity on the metal surface and to fit the Nyquist depressed semicircles more accurately. The calculated impedance parameters from the equivalent circuit are collected in Table 2. The inhibition efficiency *η*(%) of the tested inhibitors and *θ* the surface coverage was calculated using the given equations (10) and (11) [8].

(10) $$\eta \;(\text{\%})=\frac{Rp_{inh}-Rp_{blanck}}{Rp_{inh}}\times 100$$

(11) $$\theta =\frac{Rp_{inh}-Rp_{blanck}}{Rp_{inh}}$$

In this case, $R_{p}=R_{f}+R_{ct}$, and $Rp_{\mathrm{blanck}}$, $Rp_{\mathrm{inh}}$ respectively represent the polarization resistance without and with the inhibitor. The $Z_{\mathrm{CPE}}$ is expressed by the following equation (12) [8]:

(12) $$Z_{CPE}=Q^{-1}{\left(i\omega \right)}^{-n}$$

In the above equation, *Q* is the amplitude comparable to a capacity, $i^{2}=-1$, *ω* the angular frequency and n is the inhomogeneity parameter (−1<n<1) where n = 1 and n = −1 the *CPE* corresponds, respectively to a capacitor and an inductance, while if n=0 the *CPE* is equivalent to a pure resistance and when n=0.5 refers to the Warburg impedance [8].

**Table 2:** EIS data for corrosion of CS in 1M HCl without and with the different concentrations of SSBs at 308 K.

Conc.	*R*_*s*_	*R*_*f*_	*R*_ct_	*R*_*p*_	CPE	*C*_dl_	*η*
(mM)	(Ω.cm^2^)	(Ω.cm^2^)	(Ω.cm^2^)	(Ω.cm^2^)	*n*	(*μ*F.cm^−2^)	(%)
**Blank**	1.95	-	9.04	9.04	0.63	836.6	−
**SSBO**							
**0.05**	2.29	4.36	33.14	37.50	0.64	303.51	75.89
**0.10**	2.19	4.73	37.81	42.54	0.68	266.03	78.75
**0.50**	2.56	4.63	49.55	54.18	0.61	202.99	83.31
**1.00**	2.99	4.80	56.80	61.60	0.62	176.98	85.32
**SSBM**							
**0.05**	2.49	6.60	71.02	77.62	0.75	140.36	88.35
**0.10**	2.58	7.42	81.66	89.08	0.73	122.74	89.85
**0.50**	2.50	8.50	93.77	102.27	0.76	107.26	91.16
**1.00**	2.10	10.21	119.93	130.14	0.78	83.87	93.04
**SSBP**							
**0.05**	2.16	3.28	44.91	48.19	0.71	223.97	81.24
**0.1**	2.10	3.87	55.30	59.17	0.75	181.89	84.72
**0.5**	2.58	4.46	68.28	72.74	0.74	147.31	87.57
**1.00**	2.34	5.30	72.77	78.07	0.77	138.22	88.42

The capacity of the double layer (*Cdl*) was given by the following relationship (13) [10]:

(13) $$C_{dl}={\left(Q.R_{p}^{1-n}\right)}^{1/n}$$

Table 2 shows an increase in film resistance and charge transfer resistance with increasing inhibitor concentration, inducing the increase of polarization resistance (*Rp*) values and inhibitory efficiency. The increase in film strength $R_{f}$ reveals the formation of a thin film by adsorption on the metal surface. The formed film can be considered as a physical barrier that protects the CS surface from corrosion [8]. This is manifested, on the one hand, in the increase of the charge transfer resistance $R_{ct}$ and, on the other hand, in the decrease of the element *Q* and the double layer capacity $C_{dl}$. This is explained by the increase of the thickness of the organic deposit with the adsorption of the inhibitor molecules; hence the decrease of the capacity of the double layer $C_{dl}$. Knowing that, the $C_{dl}$ is inversely proportional with the organic deposition thickness by the Helmotz relation (14) [8].

(14) $$C_{dl}=\frac{\varepsilon .\varepsilon_{0}}{d}S$$

where *d* is the thickness of the protective layer, *ε* dielectric constant of the medium, $\varepsilon_{0}$ the vacuum permittivity, and *S* is the effective surface area of the electrode [10].

According to Table 2, the addition of inhibitor leads to a noticeable increase of the inhomogeneity parameter “*n*” for two inhibitors **SSBM** and **SSBP**. Similarly, by the addition of the **SSBO** inhibitor, the n parameter increases slowly compared to the n values obtained for the white solution. This result indicates that the CS surface homogeneity improved significantly by the addition of **SSBM** than **SSBP** and **SSBO**. However, even though the three tested compounds present acceptable inhibitive efficiencies, we can note that in the case of the ortho- and para-positions of the nitro group, the corresponding compounds (**SSBO** and **SSBP**) have relatively low inhibition efficiency compared to that where the nitro group in the meta-position (**SSBM**). As previously described, this could be attributed to the hydrogen bonding C-H⋯N and *π*-bonds interactions in the case of meta-position whereas the three-dimensional distance is not helpful to the formation of hydrogen bonding and/or *π*-bonds interactions in the case of ortho- and para-positions [13].

### 3.2 Stationary measurements

**Figure 5:**
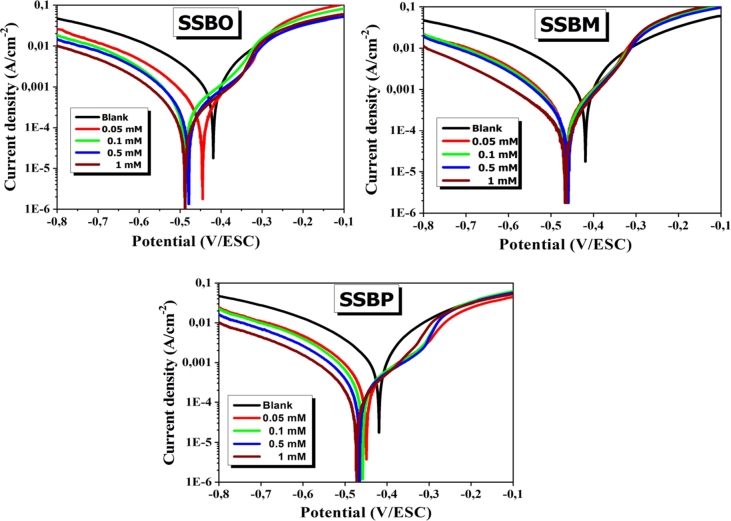
Polarization curves of CS in 1M HCl at various concentrations of **SSBs** at 308 K.

Polarization plots of CS in 1M HCl without and with inhibitor at different concentrations are given in Fig. 5. PDP data such as corrosion potential ($E_{\mathrm{corr}}$), corrosion current density ($i_{\mathrm{corr}}$), Tafel coefficients cathodic ($\beta_{c}$) and anodic ($\beta_{a}$) and inhibition efficiency *η* (%) are gathered in Table 3. The inhibition efficiency of *η* (%) was calculated according to the following equation (15) [8]:

(15) $$\eta \;(\text{\%})=\frac{i_{corr}-i_{corr}^{inh}}{i_{corr}}\times 100$$

where $i_{\mathrm{corr}}$ and $i_{corr}^{inh}$ are the corrosion current density without and with inhibitor, respectively.

**Table 3:** PDP data for CS in 1M HCl at different concentrations of studied inhibitors SSBs at 308 K.

*C*	*E*_corr_	*i*_corr_	*β*_*c*_	*β*_*a*_	*η*
(mM)	(mV/Ag-AgCl)	(*μ*A/cm^2^)	(mV/dec)	(mV/dec)	(%)
**0**	−419	879.6	−148	93	−
**SSBO**					
**0.05**	−488	321.60	−110.80	156.10	63.45
**0.10**	−487	280.15	−106.40	160.50	68.15
**0.50**	−489	205.39	−106.50	134.50	76.65
**1.00**	−498	175.48	−107.20	106	80.05
**SSBM**					
**0.05**	−466	241.90	−101.90	102.10	72.50
**0.10**	−465	209.10	−100.60	96.70	76.23
**0.50**	−461	174.90	−105.90	89	80.12
**1.00**	−466	118.20	−129.70	86.20	86.56
**SSBP**					
**0.05**	−459	257.90	−102.50	106.00	70.68
**0.10**	−475	229.03	−84.70	126.10	73.96
**0.50**	−480	177.56	−106.30	142.10	79.81
**1.00**	−488	147.33	−102.30	132.60	83.25

The addition of the inhibitors **SSBs** leads to a remarkable decrease in the current density with a displacement of the corrosion potential to negative values for all concentrations with a maximum shift not exceeding ±85 mV. This result indicates that the **SSBs** molecules behave more towards the reactive cathodic sites by delaying the reduction reaction of H+ ions. The anode branch is also affected by the addition of **SSBs** but to a lesser extent, which suggests that the **SSBs** molecules could also reduce the anodic dissolution of CS [8]. This behavior could be explained by the adsorption of **SSBs** molecules on CS surface and inhibits the dissolution of this metal.

### 3.3 Adsorption isotherm

Surface coverage *θ* for different concentrations of inhibitor are graphically tested to allow fitting of a proper adsorption isotherm. Some isotherms (Temkin, Frumkin and Langmuir isotherm) have been tested [37]. The results indicate that Langmuir isotherm is the best model that expresses the adsorption behavior of the studied interfaces. The Langmuir adsorption isotherm is given by the following equation (16) [37]:

(16) $$\frac{C_{inh}}{\theta }=\frac{1}{K_{ads}}+C_{inh}$$

where C_inh_, *θ*, and K_ads_ are the inhibitor concentration, surface coverage degree, and the equilibrium constant of adsorption process, respectively.

**Figure 6:**
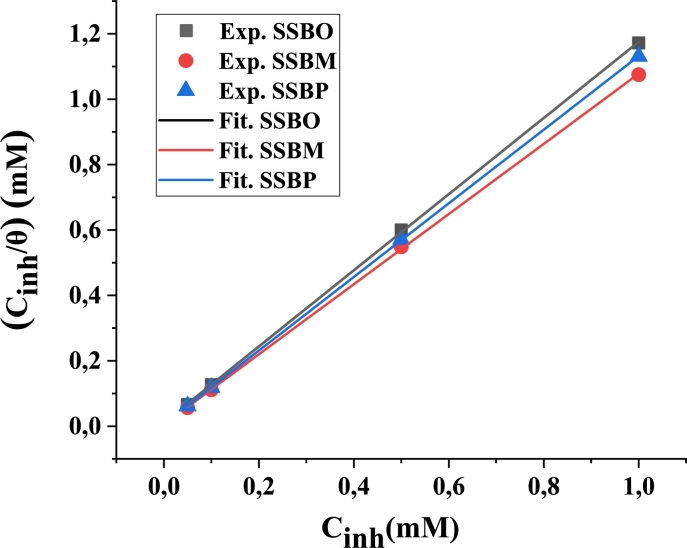
Adsorption isotherm for **SSBs** on CS surface.

C_inh_/*θ* plotted versus C_inh_ for **SSBs** which gives a straight line **(**Fig. 6).

Free energy of adsorption $\Delta G_{\mathrm{ads}}$ can be calculated as follows (17):

(17) $$\Delta G_{\mathrm{ads}}=-RT.ln(55.5K_{\mathrm{ads}})$$

with R is the perfect gas constant equal 8.314 J.mol^−1^.K^−1^, T is the temperature equal 308 K and K_ads_ is the inverse of the intercept of the plot. The decimal number 55.5 defines the concentration of water in mol.L^−1^ [38].

The values of $\Delta G_{\mathrm{ads}}$ and $K_{\mathrm{ads}}$ are grouped in Table 4.

**Table 4:** Values of K_ads_ and ΔG_ads_ for adsorption of SSBs on CS surface in 1M HCl at 308 K.

Inhibitor	*K*_ads_ (10^4^L.mol^−1^)	Δ*G*_ads_ (kJ.mol^−1^)
**SSBO**	3.730	−36.052
**SSBM**	11.038	−38.741
**SSBP**	9.615	−38.399

The calculated values of ΔG_ads_ are −36.052, −38.741 and −38.399 kJ.mol^−1^ for **SSBO**, **SSBM** and **SSBP**, respectively. Generally, the large negative extent of ΔG_ads_ showed the adsorption progress and strength of protective layer on CS surface [37]. Values of ΔG_ads_ around −20 kJ.mol^−1^ are reliable with physisorption and those around −40 kJ.mol^−1^ or higher are associated with chemisorption which due to the sharing and/or electron density transfer between organic molecules of inhibitor and CS metal [39]. In this case, the calculated ΔG_ads_ values for **SSBs** are ranging between −36.052 and −38.741 kJ.mol^−1^, suggesting that the adsorption of **SSBs** on CS surface in 1M HCl solution is both physisorption (electrostatic interactions) and chemisorption (strong interactions) [40]. Actually, it can be concluded that **SSBs** can adsorb on the CS surface in two different manners: (i) The **SSBs** molecules electrostatically adsorb onto the anions enclosed CS surface via its protonated form (ii) the **SSBs** molecules contend with chloride ions for places at the water surrounded CS surface and the unbounded electron density of nitrogen and oxygen atoms may be reacted with the empty-d orbitals of C38 steel to form a barrier film against corrosion.

### 3.4 Surface examination

**Figure 7:**
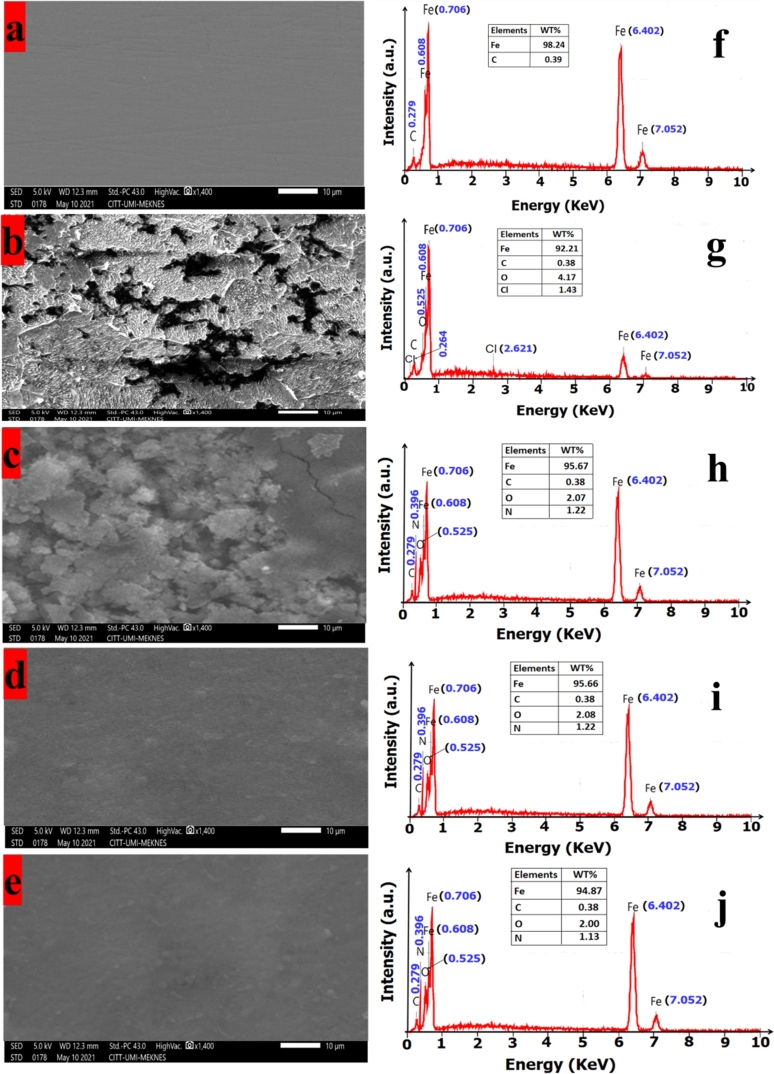
SEM images and EDS spectra for CS surfaces: (a, f) abraded, (b, g) after immersion in 1 M HCl, (c, h) after immersion in 1 M HCl +1mM of **SSBO**, (d, i) after immersion in 1 M HCl + 1mM of **SSBM**, (e, j) after immersion in 1 M HCl + 1mM of **SSBP** at 308 K.

**Figure 8:**
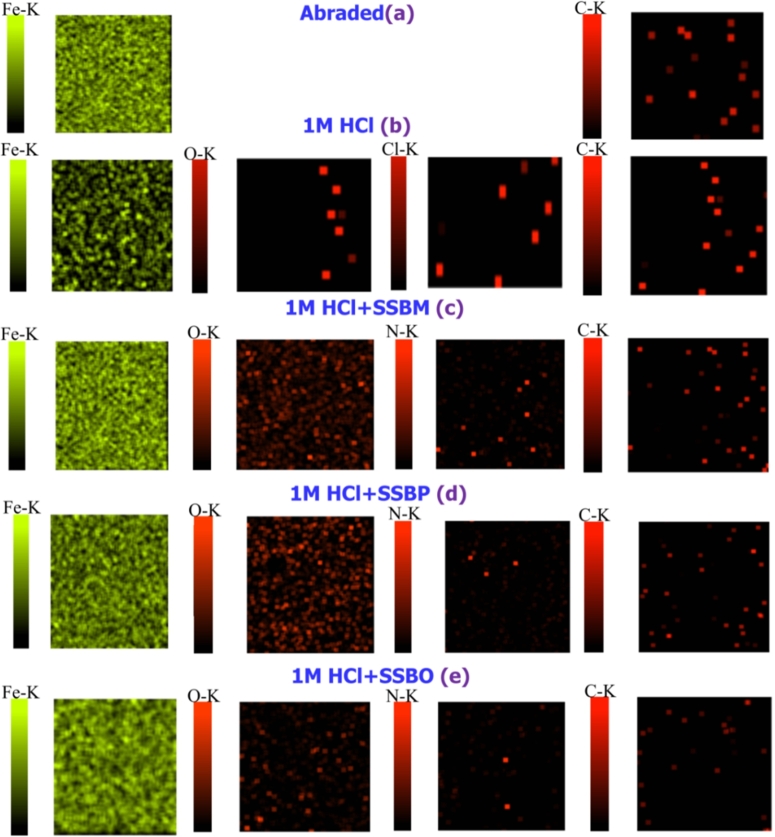
Mapping analysis for CS surfaces: (a) abraded, (b) after immersion in 1 M HCl, (c) after immersion in 1 M HCl + 1mM of **SSBM**, (d) after immersion in 1 M HCl + 1mM of **SSBP**, (e) after immersion in 1 M HCl + 1mM of **SSBO**. Green color: Fe atoms; Red color: Cl, O, N and carbon atoms.

Fig. 7a–7e displays respectively the SEM image of abraded CS surface only, in the corrosive media (1M HCl solution) and in the presence of inhibitor at the optimum concentration (1mM). When the CS surface is in contact with HCl solution, it can be scratched. The CS surface became scratched when it is in contact with HCl solution (Fig. 7b). In contrast, the CS surface remained undamaged noticeably by the addition of two inhibitors **SSBM** and **SSBP** as shown in Fig. 7c and 7d, respectively. This result can be attributed to the high capability of these inhibitors to form a better protective film entire CS surface that prohibits the migration of ionic liquid (H_2_O, Cl^−^) to the surface. Further, we noticed that **SSBO** is not contributing more to the enhancement of CS surface with respect to the **SSBM** and **SSBP**. As for **SSBP**, in spite of it improves the corroded surface of CS, its efficiency remains less compared to that of **SSBM**. So, henceforth the addition of **SSBM** inhibitor would be the real solution to protect CS surface from acidic corrosion. The presence of some elements that adhered CS surface was investigated using EDS (Fig. 7f–7j) and element mapping analysis (Fig. 8a–8d). Furthermore, it might be gotten that EDS spectra obtained of CS in corrosive media (1M HCl) shown the presence of some atoms such as iron (92.21%), carbon (0.38%), oxygen (4.17%), and chlorine (1.43%). This result shows clearly the decrease in the amount of iron and apparition of new oxygen atoms (4.17%) and chlorine (1.43%) on the uninhibited CS surface which indicates its attachment with acidic solution content; and thus the dissolution of a part of iron in acidic solution. This is in accordance with the observations investigated from element mapping analysis (Fig. 8a–8b). However, the addition of the **SSBs** does not vary the WT% of carbon obtained for abraded CS surface; in contrast, we noticed an apparition of oxygen atoms with a low weight percentage (1.22 W% for **SSBM** and **SSBP** and 1.13% for **SSBO**) compared to that obtained for uninhibited CS surface and new atoms of nitrogen with 1.22% for **SSBM** and **SSBP** and 1.13% for **SSBO**. It is also noted that the WT% of carbon atoms is almost unchanged by the addition of the inhibitor. So far, the confirmation of which inhibitor atoms can adhere to the CS surface seems unclear across EDS spectra. According to the element mapping graphs, we noticed that the number of oxygen atoms is highly increased when the inhibitor was added; while for the carbon atoms is slightly increased. As well we perceived an important amount of nitrogen atoms adhere to the CS surface. This result suggests that inhibitor molecules interact with the CS surface with their oxygen atoms of nitro groups and nitrogen atoms of 2,3-diaza group to form a protective film on the fully concerned surface. Additionally, we noticed well that the iron mapping obtained by the addition of **SSBM** and **SSBP** are almost the same as abraded CS surface. Similarly, we noticed that the addition of **SSBO** does not regenerate perfectly the iron mapping image of abraded CS surface that is considered as a reference sample. This confirms the follow trend of inhibition efficiency: **SSBM** > **SSBP** > **SSBO**.

### 3.5 DFT and QTAIM calculations

#### 3.5.1 Global reactivity behavior (GRB)

**Figure 9:**
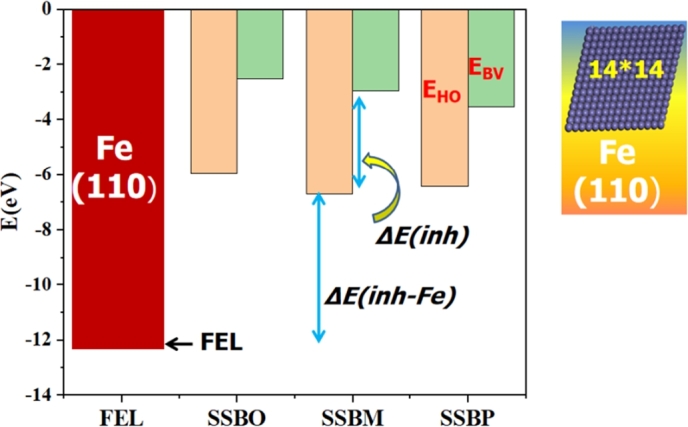
E_HO_ and E_BV_ of **SSBs** and FEL of bulk iron surface (110).

It is commonly recognized that the interaction between inhibitor and metal surface depends on the E_HO_ and E_BV_ levels of inhibitor molecule and the FEL of metal. The studied **SSBs** molecules differ from one another by the presence of the nitro group in different positions (ortho, meta or para), which probably change the electronic properties of these molecules. E_HO_ and E_BV_ for **SSBs**, and FEL for bulk iron surface (110) (supercell of 14*14) are shown in Fig. 9. Table 5 exposes the QCDs descriptors like E_HO_, E_BV_, FEL, ΔE_(inh)_, ΔE_(inh-Fe)_, *μ*, *η*, *σ* and ΔN. Except ΔE_(inh-Fe)_ descriptor, all other descriptors have been investigated the following trend of the corrosion inhibition efficiency as: **SSBP** < **SSBO** < **SSBM**, which is in disagreement with the experimental results. Otherwise, it must be noticed that some researchers discovered that ΔE_(inh)_ descriptor cannot forthrightly predict the experimental order of inhibitive performance related to the inhibitor molecules [17]. As for ΔE_(inh-Fe)_, its low value causes the growth of inhibitor molecules on the metal [17]. The calculated ΔE_(inh-Fe)_ values are 6.207, 5.485 and 5.745 eV for **SSBO**, **SSBM**, and **SSBP**, respectively. In this case, the trend of the corrosion inhibition efficiency (**SSBM** > **SSBP** > **SSBO**) for the three studied inhibitors is well in agreement with its inhibitory efficiency observed experimentally. Furthermore, electron-donating and or electron-accepting capability related to these inhibitors can also be evaluated based on the fraction of electrons transferred (ΔN) between metal substrate and inhibitor molecules (Table 5). In fact, it was pointed out that the value of ΔN is positive and less than 3.6 indicates that the studied inhibitors act as electron donors.

**Table 5:** Calculated of QCDs descriptors.

	**SSBO**	**SSBM**	**SSBP**
**E**_**HO(inh)**_**(eV)**	−5.976	−6.698	−6.438
**E**_**BV(inh)**_**(eV)**	−2.524	−2.965	−3.529
Δ**E**_**(inh)**_**(eV)**	3.452	3.733	2.909
Δ**E**_**(inh-Fe)**_**(eV)**	6.207	5.485	5.745
*μ***(eV)**	−4.250	−4.832	−4.984
*η***(eV)**	3.452	3.733	2.909
*σ***(eV**${}^{\text{-1}}$**)**	0.289	0.267	0.343
*ω***(eV)**	2.616	3.127	4.269
Δ**N**	1.149	0.984	1.237

$\text{FEL}=\mu_{Fe(110)}=-\chi_{Fe(110)}=\frac{E_{HO(Fe\left(110\right))}+E_{BV\left(Fe\left(110\right)\right)}}{2}=-12.183$ eV; $\eta_{Fe(110)}=0$ eV.

#### 3.5.2 Local reactivity behavior (LRB)

**Figure 10:**
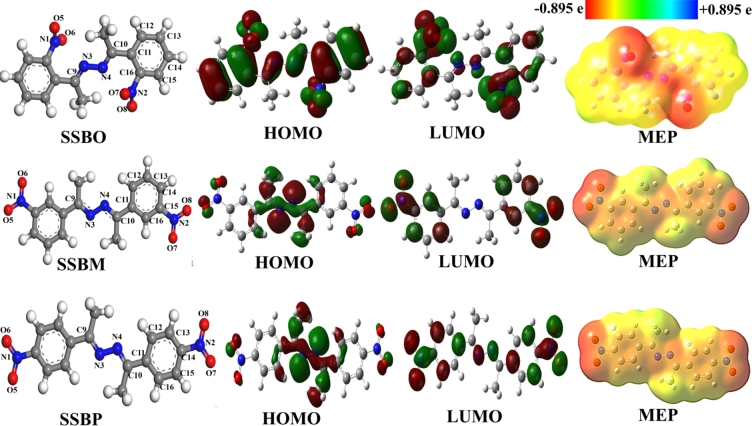
Optimized structures, HOMO/LUMO shapes and MEPs of **SSBs**. For MEP shapes, the electron density decreases in the following order: red > orange > yellow > green > blue.

**Figure 11:**
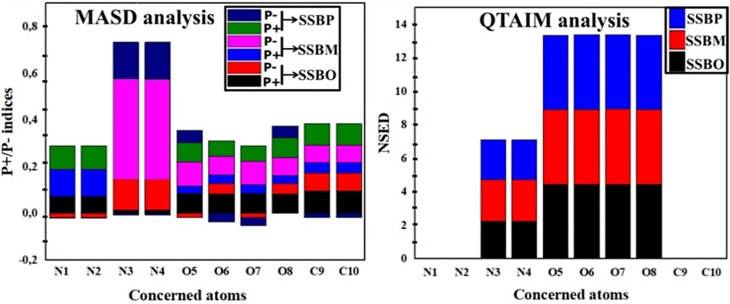
Schematic illustration of MASD and QTAIM analysis for main atoms of **SSBs**.

To determine the active centers of the inhibitor molecules, nucleophilic Parr functions *P*− (electrophilic attack) and *P*+ (nucleophilic attack), QTAIM indices, HOMO/LUMO isosurfaces and molecular electrostatic potential surface (MEP), as well as local dual descriptors like dual Fukui ($\Delta f_{k}$), dual local softness ($\Delta \sigma_{k}$) and the dual local philicity ($\Delta \omega_{k}$) were considered and discussed. Moreover, QTAIM calculations give a clear view regarding e-poor and e-rich sites of a molecule. This calculation aimed to describe non-shared electron density (NESD) around atomic site of inhibitor molecule based on the delocalized and localized index measurements. So, regarding the adsorption process, atomic sites on a molecule with a high value of *P*+ or enough value of NESD behave like nucleophilic atomic sites when they react with iron atoms' surface to form covalent bonds. Also, atomic sites with a high value of *P*− or not enough value of NESD may be responsible for forming coordination bonds by accepting electron density from the metal. Generally, the atomic centers with a negative or negligible value of Parr indices are considered as not active centers. QTAIM analysis is widely exploited to make clear the non-covalent interatomic interactions in a molecular system, by calculating the electron density that circulates within the molecular system. The analysis of HOMO/LUMO and MEP maps was performed and given in Fig. 10. Parr and QTAIM indices calculated for main atoms of the **SSBs** (Table 6 and Fig. 11).

**Table 6:** P+ and P- indices, NSED (in e) values for principal atoms of **SSBs**.

	**SSBO**			**SSBM**			**SSBP**		
No.	P+	P-	NSED	P+	P-	NSED	P+	P-	NSED
**N1**	0.073	−0.017	ED≈0	0.117	−0.001	ED≈0	0.101	−0.002	ED≈0
**N2**	0.073	−0.017	ED≈0	0.117	−0.001	ED≈0	0.101	−0.002	ED≈0
**N3**	0.014	0.112	2.254	0.006	0.401	2.484	0.009	0.157	2.372
**N4**	0.014	0.112	2.254	0.006	0.401	2.477	0.009	0.157	2.372
**O5**	0.085	−0.016	4.430	0.033	0.102	4.479	0.083	0.052	4.458
**O6**	0.084	0.045	4.455	0.037	0.078	4.472	0.067	−0.036	4.462
**O7**	0.085	−0.016	4.455	0.037	0.102	4.479	0.067	−0.036	4.458
**O8**	0.084	0.045	4.431	0.033	0.078	4.472	0.083	0.052	4.462
**C9**	0.096	0.077	ED≈0	0.046	0.074	ED≈0	0.091	−0.018	ED≈0
**C10**	0.096	0.077	ED≈0	0.046	0.074	ED≈0	0.091	0.018	ED≈0

ED: electron-deficit center.

Based on the QTAIM analysis results, we noticed for three studied inhibitors a high electron-donor activity of nitrogen atoms N3 and N4 (very enough values of NSED >2e) and oxygen atoms O5, O6, O7, and O8 (very enough values of NSED >4e). This result is confirmed by MEP plots analysis which presents strong electron density at these atoms (regions with red color) (Fig. 10). These atomic centers act as electron-donor centers when they interact with the empty-3d orbitals of the iron surface to form coordinate bonds. On the other hand, some carbon atoms of the aromatic ring and methyl groups, and some hydrogen atoms that carry low negative electron densities (regions with yellow color) are also attacked by electrophilic centers through electrostatic interactions. According to Fig. 10, we have observed also that the electronic density (HOMO) is very located on 2,3-diaza group containing nitrogen atoms (N3 and N4) for the two inhibitors (**SSBM** and **SSBP**) and on the nitro groups for the **SSBO**. While, for the electronic densities (LUMO) are low located on both aromatic ring surface and nitro groups for the three studied inhibitors. This indicates that these inhibitors have the high ability to give electrons to the metal surface (high density of HOMO) and low capability to accept electrons from the metal surface (low density of LUMO). Regarding the present case of adsorption, the high value of *P*− on any atom present in the inhibitor molecule indicates that the atom prefers to coordinate with the iron surface according to the electron-donating process. As can be observed in Table 6 and Fig. 11, *P*− values of electron-donor atoms of three studied inhibitors have increased as follows: *P*−(**SSBM**) > *P*−(**SSBP**) > *P*−(**SSBO**); this order is in parallel with inhibitory efficiency of inhibitors evidenced experimentally.

In addition, to provide more insights about local reactivity, $\Delta f_{k}$, $\Delta \sigma_{k}$ and $\Delta \omega_{k}$ are calculated. Their corresponding equations (18)–(20) are as follows [41], [42]:

(18) $$\Delta f_{k}=f_{k}^{+}-f_{k}^{-}$$

(19) $$\Delta \sigma_{k}=\sigma_{k}^{+}-\sigma_{k}^{-}$$

(20) $$\Delta \omega_{k}=\omega_{k}^{+}-\omega_{k}^{-}$$

**Figure 12:**
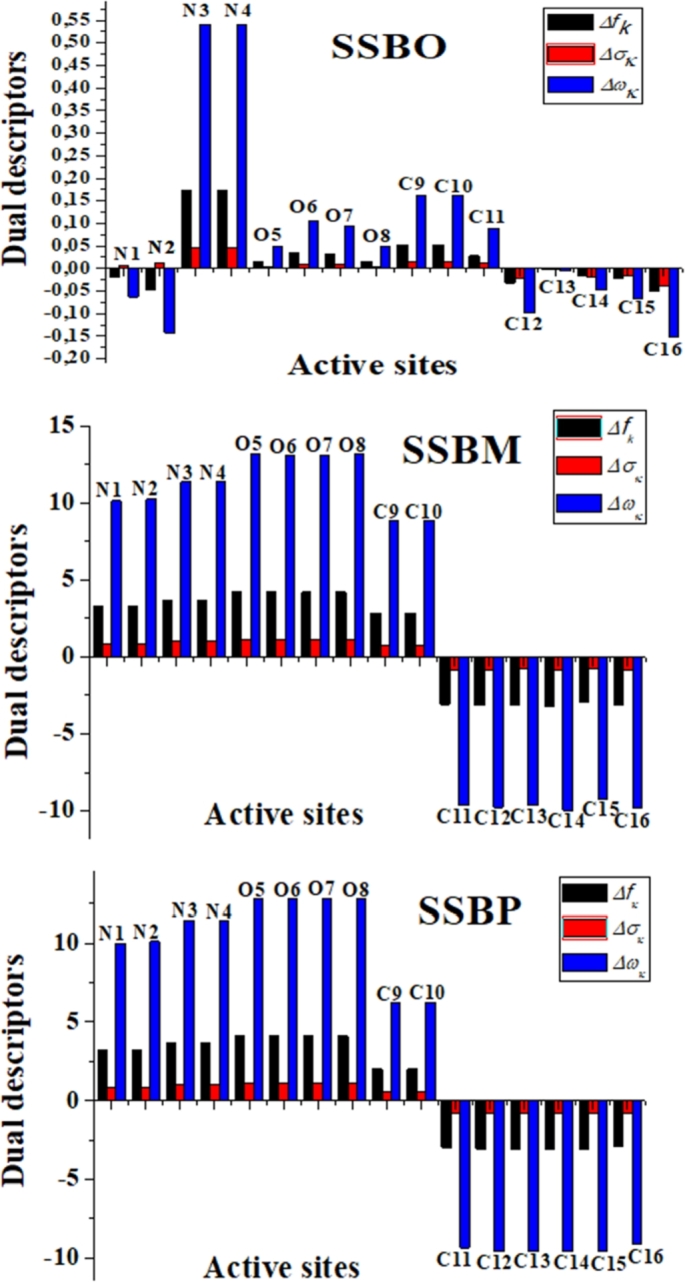
Graphical representation of the dual descriptors (Δ*f*_*k*_, Δ*σ*_*k*_ and Δ*ω*_*k*_) for the most active sites of three molecules studied **SSBs**.

It was described that if these descriptors are less than 0, the affected site is favored for an electrophilic attack. While if these descriptors are greater than 0, the affected site is favored for a nucleophilic attack. The results of Fukui functions ($f_{k}^{+}$ and $f_{k}^{-}$), the local softness ($\sigma_{k}^{+}$ and $\sigma_{k}^{-}$), the local electrophilicity ($\omega_{k}^{+}$ and $\omega_{k}^{-}$), the dual Fukui function ($\Delta f_{k}$), the dual local softness ($\Delta \sigma_{k}$), and the dual local philicity ($\Delta \omega_{k}$) are reported in Table 7. Furthermore, the results of the dual local softness and the dual local philicity are analyzed and discussed for the most active sites of molecules studied. A schematic illustration of the dual local descriptors is given in Fig. 12.

**Table 7:** Local Fukui functions, local softness, local electrophilicity, dual Fukui functions, dual local softness and dual local philicity for three studied molecules **SSBO**, **SSBM** and **SSBP**.

	No.	$f_{k}^{+}$	$f_{k}^{-}$	Δ*f*_*k*_	$\sigma_{k}^{+}$	$\sigma_{k}^{-}$	Δ*σ*_*k*_	$\omega_{k}^{+}$	$\omega_{k}^{-}$	Δ*ω*_*k*_
**SSBO**	**N1**	0.013	0.033	−0.020	0.003	0.008	0.0054	0.040	0.103	−0.063
	**N2**	−0.013	0.033	−0.046	−0.003	0.008	0.012	−0.040	0.103	−0.143
	**N3**	0.183	0.009	0.173	0.0489	0.003	0.046	0.572	0.031	0.541
	**N4**	0.183	0.009	0.173	0.049	0.003	0.046	0.572	0.031	0.541
	**O5**	0.124	0.108	0.015	0.033	0.029	0.004	0.387	0.339	0.048
	**O6**	0.136	0.102	0.034	0.036	0.027	0.009	0.425	0.319	0.106
	**O7**	0.123	0.092	0.031	0.033	0.025	0.008	0.384	0.288	0.095
	**O8**	0.099	0.084	0.015	0.026	0.022	0.004	0.309	0.262	0.048
	**C9**	0.095	0.043	0.052	0.025	0.011	0.014	0.296	0.134	0.162
	**C10**	0.095	0.043	0.052	0.025	0.011	0.014	0.296	0.134	0.162
	**C11**	0.014	−0.015	0.028	0.008	−0.004	0.011	0.044	−0.045	0.089
	**C12**	0.169	0.049	−0.032	−0.008	0.013	−0.021	0.053	0.152	−0.099
	**C13**	0.006	0.008	−0.002	−0.000	0.002	−0.003	0.020	0.025	−0.005
	**C14**	0.039	0.054	−0.015	−0.004	0.014	−0.018	0.120	0.168	−0.048
	**C15**	0.012	0.034	−0.022	−0.006	0.009	−0.015	0.037	0.105	−0.068
	**C16**	0.046	0.095	−0.049	−0.013	0.025	−0.038	0.145	0.298	−0.153
**SSBM**	**N1**	3.314	0.041	3.274	0.874	0.011	0.863	10.236	0.128	10.109
	**N2**	3.314	0.041	3.274	0.885	0.011	0.874	10.364	0.128	10.236
	**N3**	3.645	0.014	3.631	0.973	0.004	0.969	11.398	0.045	11.354
	**N4**	3.646	0.014	3.631	0.973	0.004	0.969	11.399	0.045	11.354
	**O5**	4.318	0.099	4.218	1.153	0.027	1.126	13.502	0.312	13.189
	**O6**	4.288	0.102	4.186	1.145	0.027	1.118	13.408	0.319	13.089
	**O7**	4.288	0.102	4.186	1.145	0.027	1.118	13.408	0.319	13.089
	**O8**	4.318	0.099	4.218	1.153	0.027	1.126	13.502	0.312	13.189
	**C9**	2.852	0.019	2.833	0.762	0.005	0.756	8.919	0.062	8.858
	**C10**	2.852	0.019	2.833	0.762	0.005	0.756	8.919	0.062	8.858
	**C11**	−3.044	0.033	−3.077	−0.821	0.009	−0.830	−9.517	0.101	−9.618
	**C12**	−2.979	0.143	−3.121	−0.833	0.038	−0.871	−9.314	0.446	−9.774
	**C13**	−3.118	−0.029	−3.089	−0.825	−0.008	−0.817	−9.749	−0.089	−9.660
	**C14**	−3.002	0.185	−3.187	−0.851	0.049	−0.901	−9.387	0.579	−9.966
	**C15**	−2.967	−0.006	−2.961	−0.791	−0.002	−0.789	−9.278	−0.019	−9.259
	**C16**	−3.082	0.061	−3.143	−0.839	0.016	−0.856	−9.637	0.192	−9.829
**SSBP**	**N1**	3.227	0.037	3.189	0.862	0.009	0.852	10.090	0.116	9.974
	**N2**	3.264	0.037	3.227	0.871	0.009	0.862	10.207	0.116	10.090
	**N3**	3.675	0.021	3.654	0.981	0.007	0.977	11.493	0.067	11.426
	**N4**	3.675	0.021	3.654	0.981	0.0057	0.976	11.493	0.067	11.426
	**O5**	4.199	0.104	4.096	1.121	0.028	1.094	13.132	0.324	12.809
	**O6**	4.195	0.099	4.097	1.120	0.026	1.094	13.119	0.308	12.811
	**O7**	4.196	0.100	4.096	1.120	0.027	1.094	13.122	0.314	12.809
	**O8**	4.199	0.102	4.097	1.121	0.027	1.094	13.129	0.318	12.811
	**C9**	−0.788	−2.788	2.000	−0.210	−0.744	0.534	−2.464	−8.718	6.254
	**C10**	−0.788	−2.788	2.000	−0.210	−0.744	0.534	−2.464	−8.718	6.254
	**C11**	−3.026	−0.028	−2.998	−0.800	−0.007	−0.793	−9.461	−0.087	−9.374
	**C12**	−3.029	0.047	−3.077	−0.822	0.0126	−0.834	−9.475	0.148	−9.623
	**C13**	−3.082	−0.019	−3.063	−0.818	−0.005	−0.813	−9.637	−0.059	−9.579
	**C14**	−3.007	0.061	−3.068	−0.819	0.016	−0.836	−9.402	0.192	−9.594
	**C15**	−3.238	−0.167	−3.071	−0.819	−0.0447	−0.775	−10.125	−0.523	−9.602
	**C16**	−2.811	0.122	−2.932	−0.783	0.033	−0.815	−8.789	0.381	−9.169

$\sigma_{k}^{\pm }=\sigma .f_{k}^{\pm }$; $\omega_{k}^{\pm }=\omega .f_{k}^{\pm }$.

From Table 7, the three dual local descriptors calculated for **SSBM** and **SSBP** are higher than zero for the following sites (N1, N2, N3, N4, O5, O6, O7, O8, C9 and C10), which indicate that both **SSBM** and **SSBP** have many active centers that have the ability to give electrons to the metal surface. While, it is observed that all carbon atoms of the two aromatic rings have dual local descriptors lower than zero, suggesting the presence of the electrophilic character of the active sites around the **SSBM** and **SSBP** molecules. As shown in Fig. 12, the most active sites for electron-donating centers for **SSBM** and **SSBP** have almost the same trend as follows: O5, O6, O7, O8 > N3, N4 > N1, N2. However, we noticed for these molecules that all carbon atoms of two aromatic rings have the same ability of electron-accepting character. This result is very changed for **SSBO** molecule, which presents low electrophilic sites (N1, N2 and carbon atoms of aromatic ring) and low nucleophilic sites (N3, N4, O5, O6, O7, O8, C9, C10 and C11). This confirms the previous results obtained by the analysis of the energy gap (ΔE_inh-Fe_), HOMO/LUMO shapes and QTAIM calculations.

**Figure 13:**
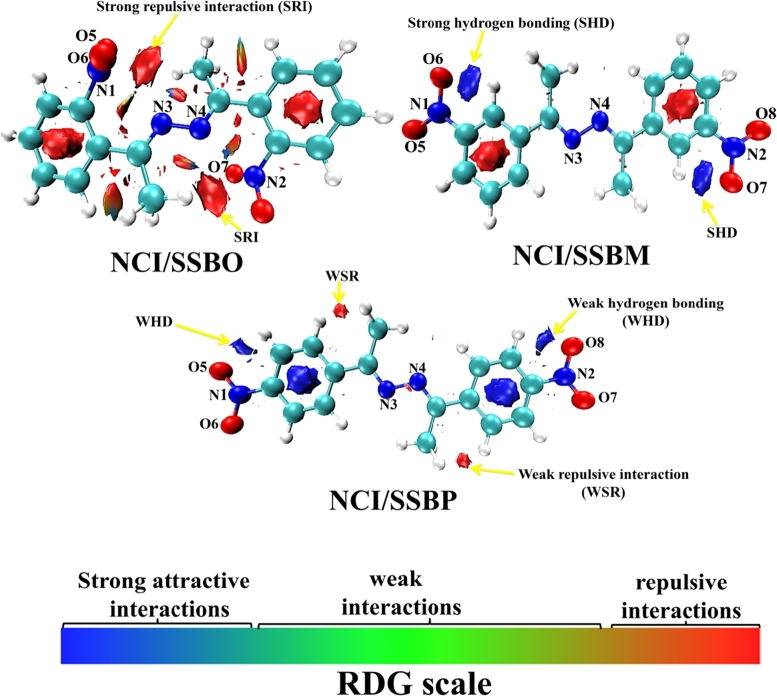
Molecular NCI plots for the studied **SSBs** molecules at a fixed reduced density gradient (RDG), *S* = 0.5 a.u., colored by the values of the sign (*λ*_2_)*ρ*[0.04, 0.02] at each point in space. RDG scale (see a legend in the bottom right) is defined as follows: Red: Strong repulsive interaction; Green: weak attractive interaction; Blue: Strong attractive interaction.

The non-covalent intramolecular interactions of **SSBs** molecules were evaluated by the molecular NCI analysis (Fig. 13). As shown in Fig. 13, we observed important repulsive interactions in almost the entire **SBBO** structure, mainly at the level of active atoms N3 and N4 of 2,3-diaza group and O5, O6, O7, and O8 of nitro groups. The presence of these repulsive interactions renders difficult the adsorption of **SSBO** with the iron surface. As for **SSBM** structure, we observed a forte attractive interaction arises between oxygen atoms of nitro groups (O6 and O7) and their adjacent hydrogen atoms of two aromatic rings, which is a sufficient condition to form hydrogen bonds in these regions and offer high stability to the **SSBM**. In addition, we noticed that this structure presents any repulsive interactions around the 2,3-diaza and the nitro groups. However, the **SSBP** structure presents a few attractive interactions that are found between oxygen atoms of nitro groups (O6 and O8) and their adjacent hydrogen atoms of two aromatic rings. Furthermore, it is noticed that **SSBP** structure also presents repulsive interactions between hydrogen atoms of methyl groups and their neighboring hydrogen atoms of two aromatic rings, implies less reactivity of **SSBP** with iron atoms compared to that of **SSBM** structure. Further to the NCI analysis, the bond critical point (BCP) analysis was performed to evaluate strength related to the hydrogen bonds that appeared at **SSBM** and **SSBP** structures using QTAIM theory. In the context of this theory, the hydrogen bonds energy ($E_{HB}$) expressed as a function of potential energy density *V*($r_{\mathrm{BCP}}$) at corresponding BCP described by the following equation (21) [43]:

(21) $$E_{HB}=\frac{V_{\left(r_{BCP}\right)}}{2}$$

The corresponding values of V(r_BCP_) related to the hydrogen bonds C_benz_-H⋯O6 and C_benz_-H⋯O7 for **SSBM**, and C_benz_-H⋯O6 and C_benz_-H⋯O8 for **SSBP** are regrouped in Table 8. From this Table, we noticed that the hydrogen bonds have more stability in the **SSBM** structure (lower value of $E_{HB}$) than in **SSBP** (higher value of $E_{HB}$); thus the stagnation of the electron density at oxygen atoms of nitro groups is more in **SSBM** than in **SSBP**, this result suggests that O-Fe interactions are more important in **SSBM** than in **SSBP** and **SSBO**.

**Table 8:** Calculated potential energy density at BCP, V(r_BCP_) in a.u., of studied hydrogen bonds (HBs) and their energy, E_HB_ in kJ.mol^−1^.

	**SSBM**			**SSBP**	
HBs	V(r_BCP_)	*E*_HB_	HBs	V(r_BCP_)	*E*_HB_
**C**_**benz**_**-H**^**...**^**O6**	−0.025	−32.468	**C**_**benz**_**-H**⋯**O6**	−0.011	−14.104
**(B3LYP)**			**(B3LYP)**		
**C**_**benz**_**-H**^**...**^**O6**	−0.027	−35.161	**C**_**benz**_**-H**⋯**O6**	−0.013	−17.013
**(B3LYP-GD3)**			**(B3LYP-GD3)**		
**C**_**benz**_**-H**⋯**O7**	−0.025	−32.468	**C**_**benz**_**-H**⋯**O8**	−0.011	−14.104
**(B3LYP)**					
**C**_**benz**_**-H**⋯**O7**	−0.027	−35.161	**C**_**benz**_**-H**⋯**O8**	−0.013	−17.013
**(B3LYP-GD3)**			**(B3LYP-GD3)**		

#### 3.5.3 Fe-complexation study

**Figure 14:**
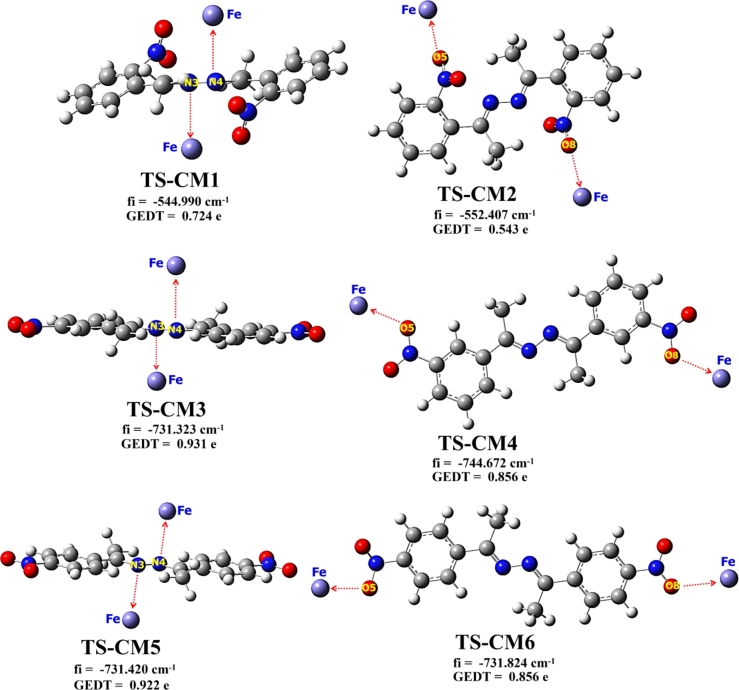
DFT/B3LYP-GD3 calculated of transition states structures for all possible complexes. fi: imaginary frequency; GEDT: global electron density transfer.

**Figure 15:**
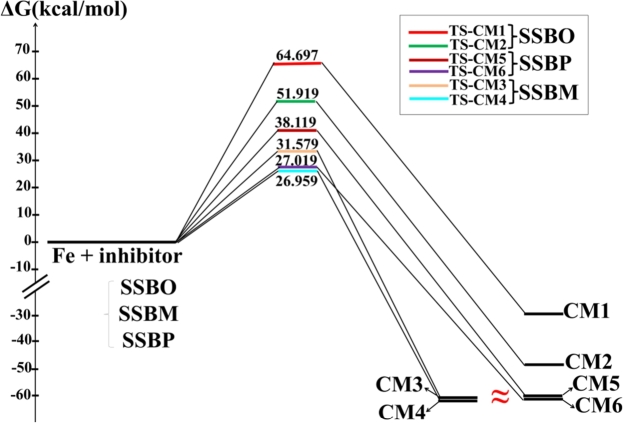
Schematic representation of free energy profile *versus* Fe-complexation coordinates.

According to QTAIM and MASD results; we can propose for each inhibitor two possibilities of complexation with Fe metal. The first one is the coordination of oxygen atoms of nitro groups with Fe, and the second is the coordination of nitrogen atoms of 2,3-diaza group with Fe. The evaluating of relative free energy (Δ*G*), activation energy (Δ*Ga*) and localization of the transition states (TSs) for the proposed complexes (Fig. 14) were assessed using QST2 approach. Table 9 shows some theoretical parameters like free energy *G*, relative free energy Δ*G* and activation energy Δ*Ga* calculated for all possible complexes structures. The Free energy profile of the Fe-complexation process is schematized in Fig. 15.

**Table 9:** B3LYP/6-311G++(2d,2p)/LanL2DZ free energy (G) and relative free energy (ΔG) and GD3-B3LYP/6-311G++(2d,2p)/LanL2DZ for activation energies (Ga) of TSs leading to the corresponding complexes.

Inhibitor	Stationary points	*G*	ΔG	ΔGa
		(a.u.)	(kcal.mol^−1^)	(kcal.mol^−1^)
**SSBO**	**SSBO+2Fe**	−612.3141672	-	−
	**CM1**	−612.3646541	−31.681	−
	**CM2**	−612.3932752	−49.641	−
	**TS-CM1 (B3LYP)**	−612.2101783	-	65.254
	**TS-CM1 (B3LYP-GD3)**	−612.2110640	-	64.697
	**TS-CM2 (B3LYP)**	−612.2309541	-	52.217
	**TS-CM2 (B3LYP-GD3)**	−612.2314285	-	51.919
**SSBM**	**SSBM+2Fe**	−723.1201421	-	−
	**CM3**	−723.2184658	−61.699	−
	**CM4**	−723.3168245	−61.721	−
	**TS-CM3 (B3LYP)**	−723.0770750	-	27.025
	**TS-CM3 (B3LYP-GD3)**	−723.0770832	-	27.019
	**TS-CM4 (B3LYP)**	−723.0698098	-	31.584
	**TS-CM4 (B3LYP-GD3)**	−723.0698174	-	31.579
**SSBP**	**SSBP+2Fe**	−805.4102132	-	−
	**CM5**	−805.5085194	−61.688	−
	**CM6**	−805.5085608	−61.714	−
	**TS-CM5 (B3LYP)**	−805.3671461	-	27.025
	**TS-CM5 (B3LYP-GD3)**	−805.3672510	-	26.959
	**TS-CM6 (B3LYP)**	−805.3494651	-	38.120
	**TS-CM6 (B3LYP-GD3)**	−805.3494651	-	38.119

According to Table 9 and Fig. 15, we noticed that the complexation was found to be spontaneous (negative value of Δ*G*). Moreover, the **SSBM** complexes present the lowest values of Δ*G* with respect to the other complexes. To this result, the thermodynamic stability of the concerned complexes can be abiding by the following trend: **SSBM**-complex > **SSBP**-complex > **SSBO**-complex. This indicates that **SSBM** has the greatest attachment and non-desorption to the iron surface with respect to the **SSBP** and **SSBO**. In addition, we noticed that the changing of nitro ortho-, meta- or para-group position causes an important change in the activation energy barrier, which are higher for **SSBO** and **SSBP**. This indicates that the coordination of **SSBM** with the iron surface is more kinetically favored (lower values of $\Delta G_{a}$) than **SSBO** and **SSBP**. This could be attributed probably to the less/hindered obstruction entire the **SSBM** structure. The GEDT values calculated for **TSs** structures and their imaginary frequencies *fi* are gathered in Table 10. Therefore, the computed GEDT at the TSs (Fig. 14) is increased in the following order: (GEDT(**TS-Fe@SSBM**) > GEDT(**TS-Fe@SSB**) > GEDT(**TS-Fe@SSB**). As conclusion, free energy, activation energy and GEDT parameters exhibit the following trend of the inhibition efficiency for the studied inhibitors: **SSBM** > **SSBP** > **SSBO**. This is in good accordance with the trend obtained experimentally.

**Table 10:** Global electronic density transfer at TSs and their imaginary frequencies.

TSs	GEDT (in e)	Imaginary frequency (cm^−1^)
**TS-CM1 (B3LYP)**	0.724	−544.991
**TS-CM1 (B3LYP-GD3)**	0.724	−544.990
**TS-CM2 (B3LYP)**	0.543	−552.406
**TS-CM2 (B3LYP-GD3)**	0.543	−552.407
**TS-CM3 (B3LYP)**	0.931	−731.325
**TS-CM3 (B3LYP-GD3)**	0.931	−731.323
**TS-CM4 (B3LYP)**	0.856	−744.671
**TS-CM4 (B3LYP-GD3)**	0.856	−744.672
**TS-CM5 (B3LYP)**	0.922	−731.418
**TS-CM5 (B3LYP-GD3)**	0.922	−731.420
**TS-CM6 (B3LYP)**	0.814	−731.825
**TS-CM6 (B3LYP-GD3)**	0.814	−731.824

As for complex systems, the molecular NBO analysis was widely used to describe donor-acceptor interactions that occur between active sites of an organic molecule and metal by calculating the second-order stabilization energy E^(2)^ and describing electronic configurations (EC) of active sites. In this present study, the E^(2)^ parameter has been calculated for possible hyper-conjugative interactions that could be between the lone pair orbital (LP) of any active donor atom (N3, N4, O5 and O8) of inhibitor and anti-lone pair orbitals (LP*) of iron atom [15]. These interactions are noticed as LP(N3)→LP*(Fe), LP(N4)→LP*(Fe), LP(O5)→LP*(Fe), and LP(O8)→LP*(Fe). Electronic configurations (EC) for atoms Fe, N3, N4, O5, and O8 have been analyzed. EC and E^(2)^ results are grouped in Table 10. Generally, a large value of E^(2)^ means a more intensive donor-acceptor interaction which could be considered as a good representation of the bond strength. The results of Table 11 show that the range of E^(2)^ values is 12.83 to 48.88 kcal.mol^−1^, 22.34 to 84.51 kcal.mol^−1^, and 19.00 to 74.92 kcal.mol^−1^ for **SSBO**, **SSBM** and **SSBP**, respectively. This result indicates that the strength of coordination of inhibitor molecule with iron atoms is in follow the trend: **SSBM** > **SSBP** > **SSBO**. Additionally, the CE results indicate clearly that the electron density transfer of adsorption takes place from nitrogen atoms (N3 and N4) of 2,3-diaza group and from oxygen atoms (O5 and O8) of nitro groups to the 3d-empty orbital of iron atoms. Electron density transfer from the active site of inhibitor molecule to iron atoms has been enhanced as follows: **SSBM** > **SSBP** > **SSBO**, which is in good agreement with the trend of inhibition efficiency obtained through the experiment essays.

**Table 11:** EC and E^(2)^ data calculated for Fe-complexation process.

	EC	*E*^(2)^ energy
**Fe+ON(SSBO)**^b^	Fe:[core]4s^1.51^3d^6.49^	−
	O5:[core]2s^1.74^2p^5.12^3p^0.01^	−
	O8:[core]2s^1.74^2p^5.12^3p^0.01^	−
**Fe@ON(SSBO)**^a^	Fe:[core]4s^1.50^3d^6.51^4p^0.06^	−
	O5:[core]2s^1.73^2p^4.88^3p^0.01^	LP(O5)→LP*(Fe)(42.02 kca.mol^−1^)
	O8:[core]2s^1.73^2p^4.88^3p^0.01^	LP(O8)→LP*(Fe)(48.88 kcal.mol^−1^)
**Fe+N=C(SSBO)**^b^	Fe:[core]4s^1.51^3d^6.49^	−
	N3:[core]2s^1.51^2p^4.38^3p^0.00^	−
	N4:[core]2s^1.51^2p^4.38^3p^0.01^	−
**Fe@N=C(SSBO)**^a^	Fe:[core]4s^1.51^3d^6.51^4p^0.03^	−
	N3:[core]2s^1.47^2p^4.31^3p^0.00^	LP(N3)→LP*(Fe)(12.83 kcal.mol^−1^)
	N4:[core]2s^1.47^2p^4.31^3p^0.00^	LP(N4)→LP*(Fe)(14.41 kcal.mol^−1^)
**Fe+ON(SSBM)**^b^	Fe:[core]4s^1.51^3d^6.49^	−
	O5:[core]2s^1.58^2p^5.01^3p^0.01^	−
	O8:[core]2s^1.58^2p^5.01^3p^0.01^	−
**Fe@ON(SSBM)**^a^	Fe:[core]4s^0.81^3d^6.83^4p^0.03^	−
	O5:[core]2s^1.72^2p^4.73^3p^0.01^	LP(O5)→LP*(Fe)(84.51 kcal.mol^−1^)
	O8:[core]2s^1.72^2p^4.73^3p^0.01^	LP(O8)→LP*(Fe)(83.96 kcal.mol^−1^)
**Fe+N=C(SSBM)**^b^	Fe:[core]4s^1.51^3d^6.49^	−
	N3:[core]2s^1.41^2p^5.09^3p^0.01^	−
	N4:[core]2s^1.41^2p^5.09^3p^0.01^	−
**Fe@N=C(SSBM)**^a^	Fe:[core]4s^0.41^3d^7.08^4p^0.06^	−
	N3:[core]2s^1.37^2p^4.08^3p^0.01^	LP(N3)→LP*(Fe)(23.31 kcal.mol^−1^)
	N4:[core]2s^1.37^2p^4.08^3p^0.01^	LP(N4)→LP*(Fe)(22.34 kcal.mol^−1^)
**Fe + ON(SSBP)**^b^	Fe:[core]4s^1.51^3d^6.49^	−
	O5:[core]2s^1.58^2p^5.00^3p^0.01^	−
	O8:[core]2s^1.58^2p^5.00^3p^0.01^	−
**Fe@ON(SSBP)**^a^	Fe:[core]4s^0.92^3d^6.81^4p^0.04^	−
	O5:[core]2s^1.70^2p^4.73^3p^0.01^	LP(O5)→LP*(Fe)(74.92 kcal.mol^−1^)
	O8:[core]2s^1.78^2p^4.75^3p^0.01^	LP(O8)→LP*(Fe)(73.96 kcal.mol^−1^)
**Fe+ N=C(SSBP)**^b^	Fe:[core]4s^1.51^3d^6.49^	-
	N3:[core]2s^1.42^2p^5.11^3p^0.00^	-
	N4:[core]2s^1.42^2p^5.11^3p^0.00^	-
**Fe@N=C(SSBP)**^a^	Fe:[core]4s^0.41^3d^7.05^4p^0.06^	-
	N3:[core]2s^1.35^2p^4.03^3p^0.00^	LP(N3)→LP*(Fe)(19.54 kcal.mol^−1^)
	N4:[core]2s^1.35^2p^4.05^3p^0.00^	LP(N4)→LP*(Fe)(19.00 kcal.mol^−1^)

a After complexation.

b Before complexation.

#### 3.5.4 Protonation of inhibitors

**Figure 16:**
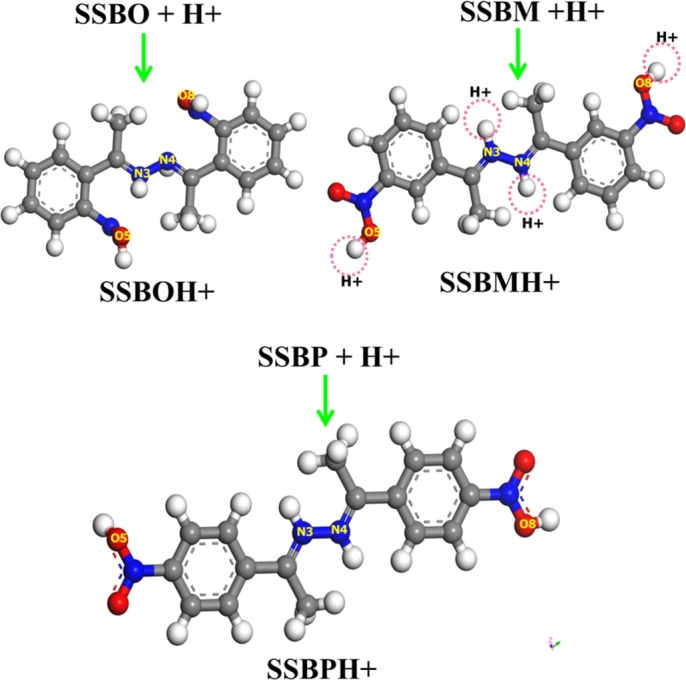
Possible protonated structures of **SSBs**→**SSBH+**.

The most nucleophilic sites of inhibitors such as N3, N4, O5 and O8 have great ability to be protonated in acidic media. This behavior was established from DFT calculations where the protonated inhibitor molecules possess lesser free energy than not protonated ones. In order to confirm possibility of protonation process of inhibitors, optimization of all possible protonated structures with different nucleophilic centers for protonation (Fig. 16) and calculating some energetic parameters were performed. Table 12 shows some theoretical parameters such as the free energy *G*, relative free energy Δ*G* and ΔE_(inh-Fe)_ calculated for all possible protonated structures. The negative value of Δ*G* showed the possibility of protonation process. Values of ΔE_(inh-Fe)_ calculated for protonated inhibitors are lower than those obtained for not protonated inhibitors. This result suggests more ability of non-protonated inhibitor molecules to adsorb iron surface compared to the protonated inhibitors.

**Table 12:** B3LYP/6-311G++(2d,2p) free energy (G), relative free energy (ΔG) and ΔE_(inh-Fe)_ of protonation process.

	G(a.u.)^a^	ΔG(kcal/mol)	ΔE_(inh-Fe)_ (eV)
**SSBO + 4H+**	−376.3758116	−15.125	−
**SSBOH+**	−367.3999148	-	7.314
**SSBM + 4H+**	−478.1817865	−22.004	−
**SSBMH+**	−478.2168521	−18.984	6.317
**SSBP + 4H+**	−590.4718576	−18.984	−
**SSBPH+**	−590.5021105	-	6.681

a 1u.a. = 627.509 kcal/mol.

### 3.6 Molecular dynamics calculations

#### 3.6.1 Strength of interfacial interaction

**Figure 17:**
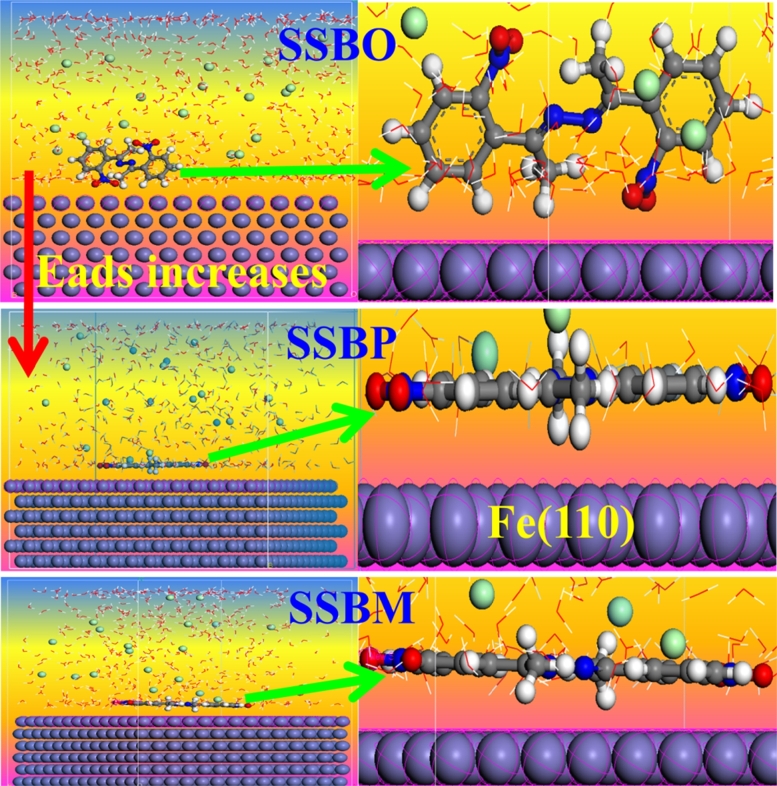
Stable adsorption configuration of **SSBs** on iron (110) surface at 308 K.

**Figure 18:**
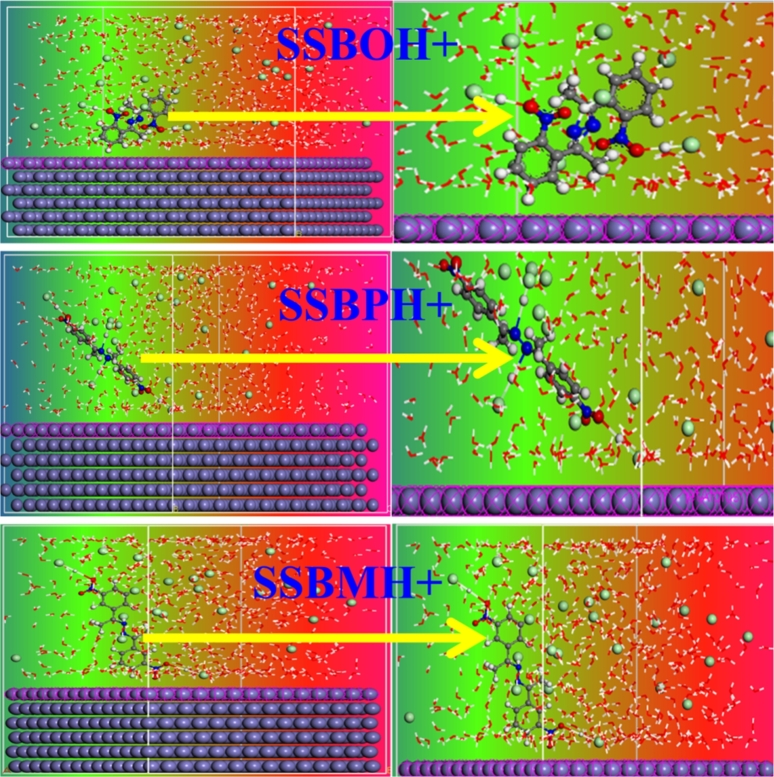
Stable adsorption configuration of **SSBH+** on iron (110) surface at 308 K.

Molecular dynamics simulation was performed at temperature of 308 Kelvin until the studied interface reaches equilibrium. In this context, the most stable adsorption configurations of the not protonated molecules (**SSBs**) and the protonated ones (**SSBH+**) on Fe (110) surface are given in Fig. 17 and 18, respectively. Besides, the strength of the interfacial interactions has generally been evaluated based on the adsorption energy ($E_{\mathrm{ads}}$) or binding energy ($E_{\mathrm{binding}}$) and desorption energy ($dE_{\mathrm{ads}}$/$dN_{\mathrm{inh}}$). Binding energy is defined as the opposite of adsorption energy as follows (22) [33]:

(22) $$E_{binding}=E_{total}-(E_{surface+solution}+E_{inhibitor})$$

In addition, $dE_{\mathrm{ads}}$/$dN_{\mathrm{inh}}$ is the energy required to remove an adsorbate from the iron surface (110), a high value of $dE_{\mathrm{ads}}$/$dN_{\mathrm{inh}}$ due to the strong adsorption of the inhibitor on the iron surface (110) [10], [44]. The high value of the binding energy (or high absolute value of the adsorption energy) reproduces strong adsorption behavior. The different values of the energies are calculated in the solution containing the inhibitor, 500 molecules of water, 20 oxonium ions (H_3_O^+^), and 20 chlorine ions Cl^−^ and reported in Table 13. From the careful observation of Fig. 17 it can be said that the molecules **SSBM** and **SSBP** adsorb in large part on the iron surface, which demonstrates that these molecules have a high tendency to form a dense film entire iron surface and consequently promoting the protection of C38 steel against corrosion in the 1M HCl solution. However, the adsorption of **SSBO** is happening in a part consisting of nitrogen dioxide and the phenyl double bond, while the rest of the molecule is taking a sloping form on the surface solution. Additionally, these observations confirm the high inhibitory efficiency of **SSBM** and **SSBP** due to the more active site over molecule structures of these inhibitors. As for the protonated forms of inhibitors (Fig. 18), we observed that all protonated inhibitors are not oriented appropriately above the iron surface, and thus leads to the low coverage of these molecule structures onto the iron surface. These results allow us to conclude that the anti-corrosive property is favorable when the inhibitors studied were at their neutral forms (non-protonated forms). Furthermore, as shown in the Table 13, the negative values of $E_{\mathrm{ads}}$ mean that the studied adsorption is spontaneously shaped and thus, the inhibitor has qualitatively a high adsorption capability to interact with the iron surface [10], [44]. Moreover, we noticed that the binding energy ($E_{\mathrm{binding}}$) related to the adsorption of inhibitors on Fe (110) surface increased in the following order: **SSBO**/Fe (110) < **SSBP**/Fe (110) < **SSBM**/Fe (110). This is in good agreement with the trend of inhibitory efficiency found in the electrochemical and quantum studies sections. This observation is strengthened by the comparison of the values of desorption energies that shows the following trend: $dE_{\mathrm{ads}}$/*dNi* (**SSBM**) > $dE_{\mathrm{ads}}$/*dNi* (**SSBP**) > $dE_{\mathrm{ads}}$/*dNi* (**SSBO**). This difference could be explained by the fact that **SSBM** and **SSBP** present less hindered/steric obstruction which gives them a high coverage entire iron surface. For the protonated forms, we noticed a strong decrease in both binding energy and desorption energies (Table 13). This result indicates lower adsorption of **SSBH+** on the iron surface than **SSBs** in 1M HCl. The results obtained were supported by other calculations discussed below based on the pair atomic distribution function (PADF), mean square displacement (MSD) and free volume fraction (FVF) [33], [41], [45].

**Table 13:** *E*_*ads*_, *E*_*binding*_ and *dE*_*ads*_/dNi energies at 308 K for protonated SSBs and unpronated SSBH+ adsorbed molecules. All energies are in kcal.mol^−1^.

	*E*_ads_	*E*_binding_	dE_ads_/dN_inh_
**SSBO/Fe(110)**	−5144.103	5144.103	−87.524
**SSBM/Fe(110)**	−7148.331	7148.331	−65.479
**SSBP/Fe(110)**	−6920.170	6920.170	−79.895
**SSBOH+/Fe(110)**	−644.008	644.008	−487.102
**SSBMH+/Fe(110)**	−1148.125	1148.125	−391.012
**SSBPH+/Fe(110)**	−1020.119	1020.119	−397.147

#### 3.6.2 PADF calculations

The pair atomic distribution function (PADF) theory, so-called g(r) function, is most commonly used to describe how the electron density between the atoms of a molecule and iron surface varies with distance from an infinitesimal distance r+dr. This theory gives information about the probability with which certain inter-atomic distances between atoms X and Y [33]. The g(r) is defined as the ratio of the local density of “Y” at a distance r from “Y” particles by the following equation (23):

(23) $$g_{XY}\left(r\right)=\frac{1}{{\langle \rho_{Y}\rangle }_{local}}x\frac{1}{N_{X}}\sum_{i\epsilon X}^{N_{X}}\sum_{j\epsilon Y}^{N_{Y}}\frac{\delta (r_{ij}-r)}{4\pi r^{2}}$$

where $\langle \rho_{Y}\rangle$_local_ represents the average local number density of “Y” particles over all layers which surround the “X” particle.

**Figure 19:**
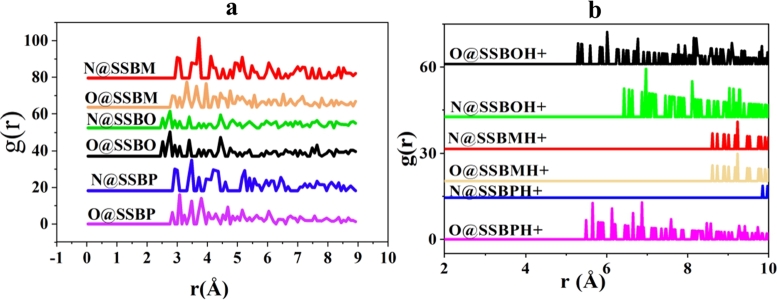
PADF analysis of (a) **SSBs** and (b) **SSBH+** on Fe (110) surface in acidic media at 308 K.

In general, if the distance values between X and Y atoms include the range [1-3.5 Å], strong connections are present (i.e. chemical bonds). If the contrary, there are weak connections (i.e. physical bonds) [33]. Fig. 19 shows the variation of PADF versus the bond lengths (r) such as Fe-N3, Fe-N4, Fe-O5, and Fe-O8 for **SSBs** and **SSBH+**. For the non-protonated forms of inhibitors (Fig. 19a), we observed the appearance of peaks with distances < 3.5 Å and the others with distances > 3.5 Å. This result indicates that **SSBO**, **SSBM**, **SSBP** coordinate to Fe (110) surface through both chemical and physical bonds. While, for the protonated forms of inhibitors (Fig. 19b), it is clearly remarked that all peaks have appeared at distances > 3.5 Å; indicating the presence of weak connections between **SSBH+** and iron surface. To these results, we concluded that adsorption of inhibitors onto the iron surface is mixed-type (chemisorption and physisorption), and the non-protonated structures of the inhibitor are dominated in the adsorption than the protonated ones.

#### 3.6.3 MSD calculations

**Figure 20:**
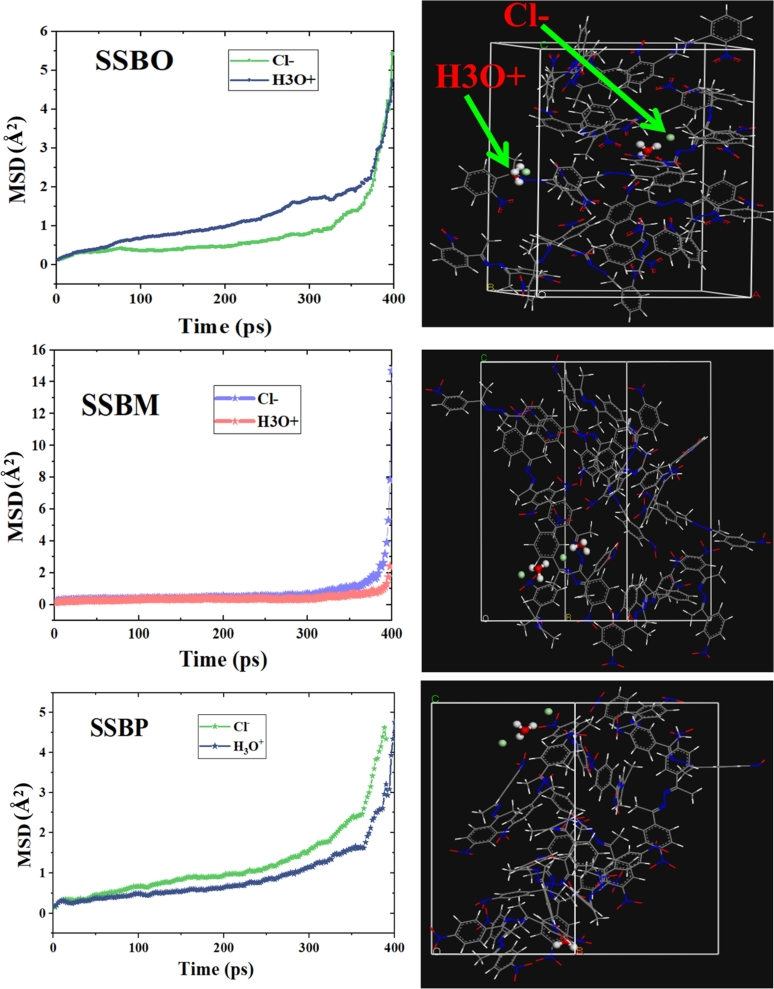
MSD plots and the diffusion coefficient of the studied ions (H_3_O^+^ and Cl^−^) in the **SBBs** molecules at 308 K.

**Figure 21:**
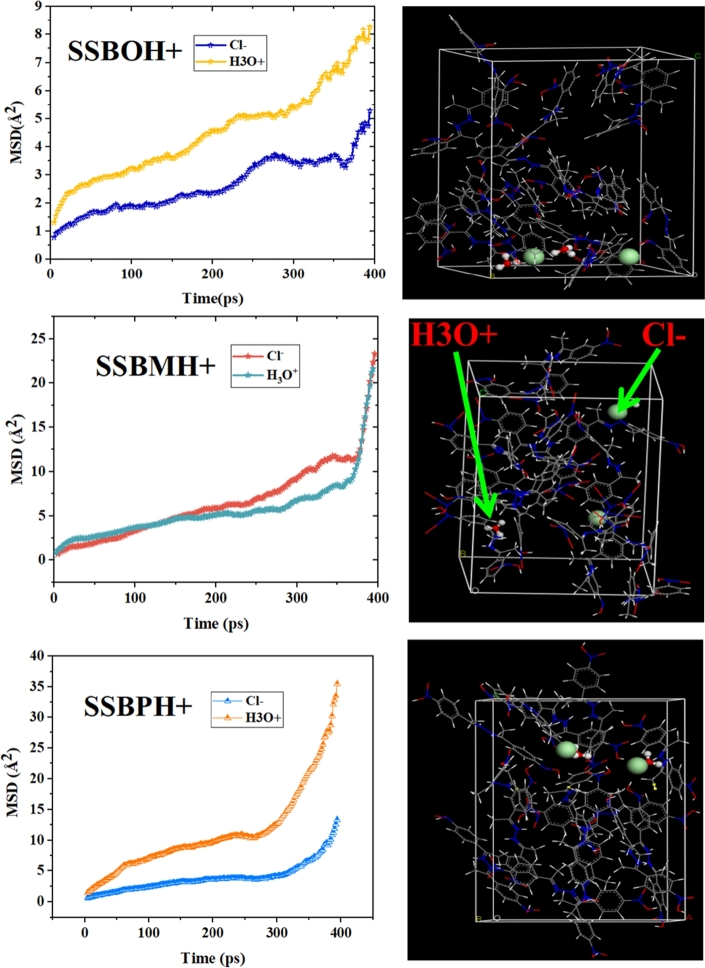
MSD curves and the diffusion coefficient of the studied ions (H_3_O^+^ and Cl^−^) in the **SBBH+** molecules at 308 K.

The mean square displacement (MSD) curves and the diffusion coefficient (D) behavior of corrosive ions (3H_3_O^+^ and 3Cl^−^) in the supercell that covers 30 molecules of each inhibitor (protonated or non-protonated one) were modeled through Einstein equations (24) and (25), respectively [45]. MSD and diffusion coefficient calculations for **SSBs** and **SSBH**${}^{\text{+}}$ are shown in Fig. 20 and 21, respectively.

(24) $$MSD=\langle |R_{i}\left(t\right)-R_{i}\left(0\right){|}^{2}\rangle$$

(25) $$D=\frac{1}{6N_{\alpha }}\lim_{x\to \infty }\frac{d}{dt}\sum_{i=1}^{N_{\alpha }}\langle |R_{i}\left(t\right)-R_{i}\left(0\right){|}^{2}\rangle$$

where *Nα* represents the number of corrosive ions, whereas $R_{i}\left(t\right)$ and $R_{i}\left(0\right)$ are the displacement of the corrosive ion between moment t and the initial moment t_0_ respectively. The diffusion coefficient can be calculated through MSD curves using the following equation (26) [45], [46]:

(26) $$D=\frac{m}{6}$$

where the m is the slope of MSD curve.

Furthermore, it is noted that a low diffusion coefficient (D) value reflects high corrosion inhibition efficiency. The D values are concluded from the RMS curves gathered in Fig. 20 and 21. According to these figures, the D values for H_3_O^+^ are 0.00454 × 10^12^, 0.00179 × 10^12^, and 0.00255 × 10^12^ m^2^/s for **SSBO**, **SSBM** and **SSBP** molecules, respectively. However for Cl^−^ the D values are 0.00423 × 10^12^, 0.00219 × 10^12^, and 0.00229 × 10^12^ m^2^/s for **SSBO**, **SSBM** and **SSBP** molecules, respectively. Moreover, the D values of Cl^−^ ions are 0.00290 × 10^12^, 0.00223 × 10^12^, and 0.00254 × 10^12^ m^2^/s for **SSBOH**+, **SSBMH+** and **SSBPH+**, respectively. Then, for H_3_O^+^ ions the D values are 0.00572 × 10^12^, 0.00337 × 10^12^, and 0.00381 × 10^12^ m^2^/s for **SSBOH+**, **SSBMH+** and **SSBPH+**, respectively. These findings suggest the best inhibition efficiency of **SSBM** (low value of D) than **SSBO** and **SSBP**, which is in good agreement with all results discussed above.

#### 3.6.4 FFV calculations

Further, the evaluation of free volume inside each inhibitor was performed by molecular dynamics simulation. Based on the outputted results of MDS, the free fractional volume (FFV), is calculated using the following equation (27) [45], [46], [47]:

(27) $$FFV=\frac{V_{free}}{V_{free}+V_{Occ}}\times 100\text{\%}$$

where V_free_ is the free volume and V_occ_ is the volume occupied by the inhibitor film on the CS surface.

**Figure 22:**
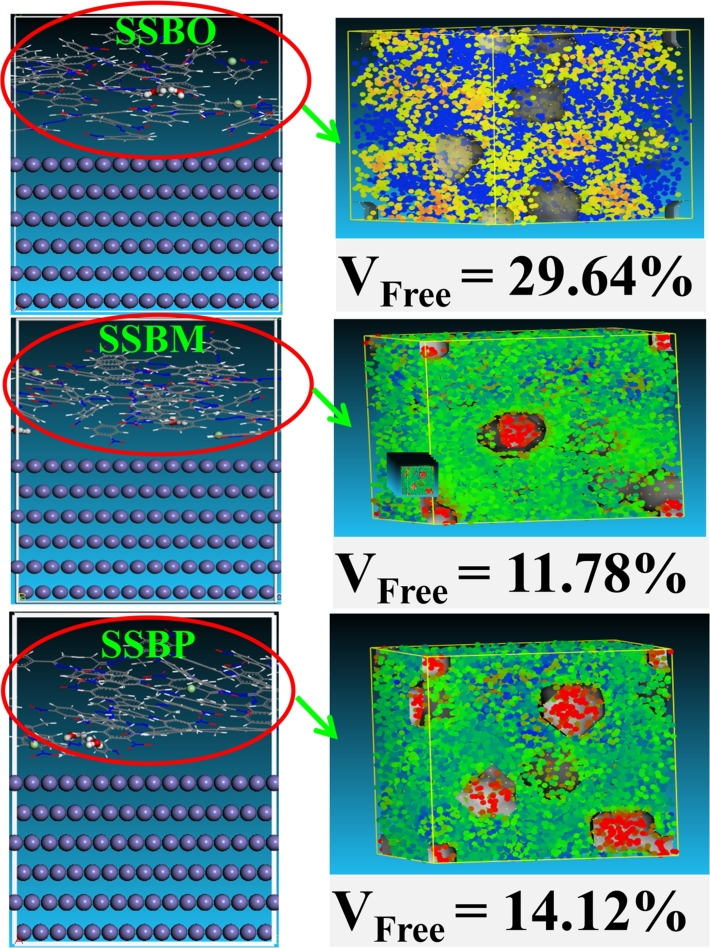
(Left) the equilibrium adsorption configurations of **SSBs** on iron (110) surfaces at 308 K; (right) the free volume distribution after 1 ns MD simulation.

V_free_ is concluded as given by the following equation (28) [47]:

(28) $$V_{free}=\frac{E_{free}}{PRT}$$

where E_free_ is the free energy density, R is the universal gas constant, T is the temperature, and P is the atmospheric pressure. H3O^+^ and Cl^−^ ions were chosen as probe particles (sphere radii 2 nm and bead diffusion coefficient e^−7^ cm^2^/s). Evidently, a large value of FFV means that there are abundant voids over inhibition film, and the movement probability of corrosive species is high, which makes to low inhibition efficiency. While, a small value of FFV induces to high inhibition efficiency of corrosion [45], [46]. As shown in Fig. 22, the molecular graphic showing the free volume distribution (blue and red regions) through an amorphous cell contains 15 inhibitors, 2H_3_O^+^ and 2Cl^−^. Indeed, red color indicates high free energy density (i.e., low free volume), the blue color indicates low free energy (i.e. high free volume), and green color represents the occupied volume by inhibitor molecules [48]. The free volume distributions and FFV values for **SSBs** molecules are given in Fig. 22. Indeed, the present FFV values calculated for **SSBO**, **SSBM** and **SSBP** are 29.64, 11.78 and 14.12%, respectively. This observation reveals that the addition of **SSBs** molecules into the corrosive environment creates a barrier film on the CS surface and limits migration of corrosive species, which is in the following order: **SSBM** > **SSBP** > **SSBO**. This result further reinforces the high inhibition performance of **SSBM** than **SSBO** and **SSBP** and supports all results discussed previously in this work.

### 3.7 Inhibitive corrosion mechanism

**Figure 23:**
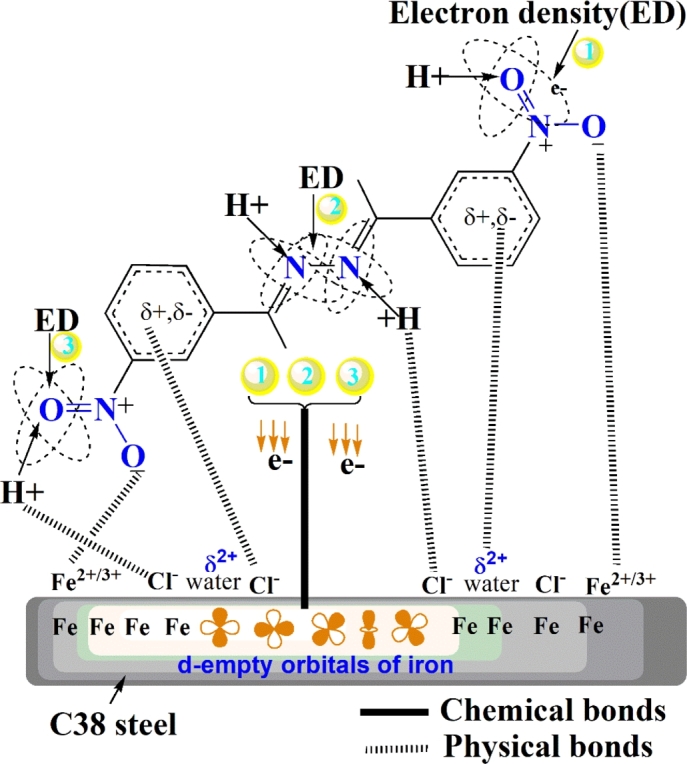
Proposed inhibitive corrosion mechanism.

**Figure 24:**
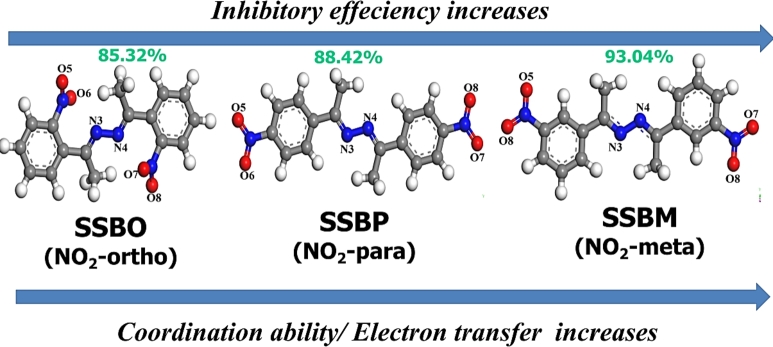
Schematic illustration of inhibitory order associated to the **SSBs** molecules.

From the results discussed above, the safeguarding of C38 steel by the presence of **SSBs** inhibitors depends on the higher electron density clouds around the nucleophilic atoms (N3, N4, O5 and O8), the *π* electrons of 2,3-diaza/nitro groups and lone-pair electrons of nitrogen and oxygen atoms (N3, N4, O5 and O8) available on **SSBs** molecules, which shared with d-empty orbitals of CS to form a protective layer against corrosion. Additionally, the **SSBs** molecules are adsorbed electrostatically onto the anions enclosed CS surface via its protonated form. The inhibitive corrosion mechanism is illustrated in Fig. 23. The order of the inhibitory efficiency for **SSBs** molecules where the nitro group places in ortho- (**SBBO/NO2-ortho**), meta- (**SSBM/NO2-meta**) or para- (**SSBP/NO2-para**) positions is illustrated in Fig. 24.

A comparison of inhibition efficiency *η* (%) with similar organic compounds shows the higher inhibitive performance of our tested inhibitors; in particular, **SSBM** can be considered a promising candidate for corrosion inhibition of C38 steel in acidic media (Table 14).

**Table 14:** Similar hydrazine derivatives as corrosion inhibitors by other authors for steel in 1 M HCl solution at 308 K.

Inhibitor	*η* (%)	Reference
(*1E,2E*)-bis(1-(2-nitrophenyl)ethylidene)hydrazine	85.32	This work
(*1E,2E*)-bis(1-(3-nitrophenyl)ethylidene)hydrazine	93.04	This work
(*1E,2E*)-bis(1-(4-nitrophenyl)ethylidene)hydrazine	88.42	This work
(*1E,2E*)-1,2-bis(thiophen-2-ylmethylene)hydrazine	62.40	[10]
(*1E,2E*)-1,2-bis(1H-pyrrol-2-ylmethylene)hydrazine	86.70	[10]
(*1E,2E*)-1,2-bis(pyrrol-2-ylidenemethyl)hydrazine	79.50	[11]
(*1E,2E*)-1,2-bis(thiophen-2-ylidenemethyl)hydrazine	85.57	[12]
(*1E,2E*)-1,2-Bis(furyl-2-ylidenmethyl)hydrazine	84.93	[12]

## 4 Conclusion

In this study, three hydrazine derivatives **SSBO**, **SSBM**, and **SSBP** have been effectively explored as cost-effective and eco-friendly inhibitors against corrosion of C38 steel in 1M HCl solution. The concluded results are mentioned below:

• The inhibition efficiency is improved when the inhibitor concentration increased to reach a maximum of 85.32, 88.42% and 93.04, at 1 mM for **SSBO**, **SSBP** and **SSBM**, respectively;
• The PDP measurements indicated that **SSBs** were a mixed-type inhibitor with a predominantly cathodic control;
• The EIS results showed that there are two capacitive loops. The first at high frequencies was attributed to the inhibitor film and the second was related to the charge transfer phenomenon;
• The adsorption isotherm study showed that the tested inhibitors obeyed the Langmuir adsorption isotherm and the ΔG_ads_ values suggested that these inhibitors acted through a chemisorption process;
• The protective effect of **SSBs** was confirmed using SEM-EDS test and element mapping analysis;
• Experimental results were successfully elucidated based on quantum chemical calculations;
• Molecular dynamics simulation showed clearly that the inhibitive performance is better for the not protonated inhibitor molecules than protonated ones;
• The inhibition efficiency is strongly depending on both the electronic structure and the nature of the geometry of the assayed compounds.

## Declarations

### Author contribution statement

Zouhair Lakbaibi, Mohamed Damej, Mohammed Benmessaoud, Adil Jaafar, Tariq Benabbouha, Mohamed Tabyaoui: Conceived and designed the experiments; Performed the experiments; Analyzed and interpreted the data; Wrote the paper.

Abdu Molhi, Said Tighadouini, Abdeselam Ansari, Anas Driouich: Contributed reagents, materials, analysis tools.

### Funding statement

This research did not receive any specific grant from funding agencies in the public, commercial, or not-for-profit sectors.

### Data availability statement

Data included in article/supplementary material/referenced in article.

### Declaration of interests statement

The authors declare no conflict of interest.

### Additional information

No additional information is available for this paper.
